# Mitochondrial Calcium Uniporter (MCU) deficiency reveals an alternate path for Ca^2+^ uptake in photoreceptor mitochondria

**DOI:** 10.1038/s41598-020-72708-x

**Published:** 2020-09-29

**Authors:** Celia M. Bisbach, Rachel A. Hutto, Deepak Poria, Whitney M. Cleghorn, Fatima Abbas, Frans Vinberg, Vladimir J. Kefalov, James B. Hurley, Susan E. Brockerhoff

**Affiliations:** 1 Biochemistry Department, University of Washington, Seattle, WA USA; 2 Department of Ophthalmology and Visual Sciences, Washington University School of Medicine, St. Louis, MO USA; 3 Ophthalmology and Visual Sciences, University of Utah, Salt Lake City, UT USA; 4 Ophthalmology Department, University of Washington, Seattle, WA USA

**Keywords:** Biochemistry, Calcium channels, Visual system, Retina, Mitochondria, Energy metabolism, Metabolism

## Abstract

Rods and cones use intracellular Ca^2+^ to regulate many functions, including phototransduction and neurotransmission. The Mitochondrial Calcium Uniporter (MCU) complex is thought to be the primary pathway for Ca^2+^ entry into mitochondria in eukaryotes. We investigate the hypothesis that mitochondrial Ca^2+^ uptake via MCU influences phototransduction and energy metabolism in photoreceptors using a *mcu*^-/-^ zebrafish and a rod photoreceptor-specific *Mcu*^*-/-*^ mouse. Using genetically encoded Ca^2+^ sensors to directly examine Ca^2+^ uptake in zebrafish cone mitochondria, we found that loss of MCU reduces but does not eliminate mitochondrial Ca^2+^ uptake. Loss of MCU does not lead to photoreceptor degeneration, mildly affects mitochondrial metabolism, and does not alter physiological responses to light, even in the absence of the Na^+^/Ca^2+^, K^+^ exchanger. Our results reveal that MCU is dispensable for vertebrate photoreceptor function, consistent with its low expression and the presence of an alternative pathway for Ca^2+^ uptake into photoreceptor mitochondria.

## Introduction

Maintaining proper intracellular Ca^2+^ homeostasis is essential for cellular function. Mitochondria have the ability to sequester Ca^2+^ via the Mitochondrial Calcium Uniporter complex (MCU)^1,2^. Mitochondrial Ca^2+^ uptake via MCU can modulate both cytosolic and mitochondrial Ca^2+^ levels, meaning that Ca^2+^-sensitive reactions that occur in both compartments can be affected by MCU activity. In the mitochondrial matrix, increasing Ca^2+^ can stimulate tricarboxylic (TCA) cycle dehydrogenases and enhance ATP production, although Ca^2+^ overload in the matrix can trigger cell death^3,4^. Mitochondrial Ca^2+^ uptake can also regulate cytosolic Ca^2+^ levels and thus influence Ca^2+^-sensitive cytosolic reactions. Since these Ca^2+^-sensitive cytosolic reactions vary widely among cell-types depending on each cell’s specific function, disruptions in mitochondrial Ca^2+^-uptake can have tissue-specific consequences^5,6^.

Photoreceptors, the light-sensitive neurons in the retina, rely on spatially distinct changes in cytosolic Ca^2+^ to regulate both phototransduction and neurotransmission. At the outer segment, intracellular Ca^2+^ changes in response to light stimulation and this is critical for timely shut-down of the phototransduction cascade and light adaptation^7^. At the synapse, changes in intracellular Ca^2+^ modulate vesicle release and neurotransmission^8–10^. The outer segment and synaptic Ca^2+^ pools that control these functions are separated by a cell body filled with a dense cluster of mitochondria, and evidence suggests that these mitochondria may help isolate these pools from each other. Mitochondria from zebrafish cones can prevent Ca^2+^ in the outer segment from reaching the rest of the cell and conversely, they can prevent Ca^2+^ in the synapse from reaching the outer segment^11^. Increasing mitochondrial Ca^2+^ uptake in zebrafish cones by overexpressing MCU also accelerates cytosolic Ca^2+^ clearance, which causes cones to recover from light exposure faster^12^.

Efficient uptake of Ca^2+^ into mitochondria could also enhance the ability of photoreceptors to meet their high energy demands. Darkness is the most energetically demanding state of the photoreceptor, and intracellular Ca^2+^ is at its highest levels in darkness^13,14^. If this Ca^2+^ were to enter mitochondria, it could enhance ATP production by stimulating TCA cycle dehydrogenases^3^. In line with this hypothesis, overexpressing MCU in zebrafish cones alters the distribution of TCA cycle metabolites in a way which is consistent with enhanced activity of Ca^2+^-sensitive dehydrogenases^12^.

These observations suggested to us that mitochondrial Ca^2+^ uptake could play an important role in modulating photoreceptor function. Given this, it is surprising that zebrafish cone photoreceptors express extremely low levels of MCU^12^. MCU has been widely thought to be the sole route of Ca^2+^ entry into mitochondria in eukaryotes, as loss of MCU completely inhibits mitochondrial Ca^2+^ uptake in skeletal muscle, liver, heart, brown adipose tissue, and a wide variety of cell lines^15–19^. Despite this, it is possible that certain specialized cell types might not rely solely on MCU for mitochondrial Ca^2+^ uptake, since it also has been observed that brain mitochondria lacking MCU expression do not have a complete loss of mitochondrial Ca^2+^ uptake^20^. Thus, the role that MCU plays in vertebrate photoreceptors remains an open question.

To resolve the role MCU-mediated mitochondrial Ca^2+^ uptake plays in modulating photoreceptor function, we evaluated the morphological, biochemical and physiological consequences of loss of MCU expression using both a global *mcu*^*-/-*^ zebrafish model and a rod photoreceptor-specific *Mcu*^-/-^ mouse model. Our study demonstrates that MCU is remarkably dispensable for photoreceptor function, which is consistent with our discoveries of very low levels of MCU expression and evidence for an additional mechanism for Ca^2+^ uptake into photoreceptor mitochondria.

## Results

### Mcu contributes to clearance of cytosolic Ca^2+^ in zebrafish cones

**Figure 1:**
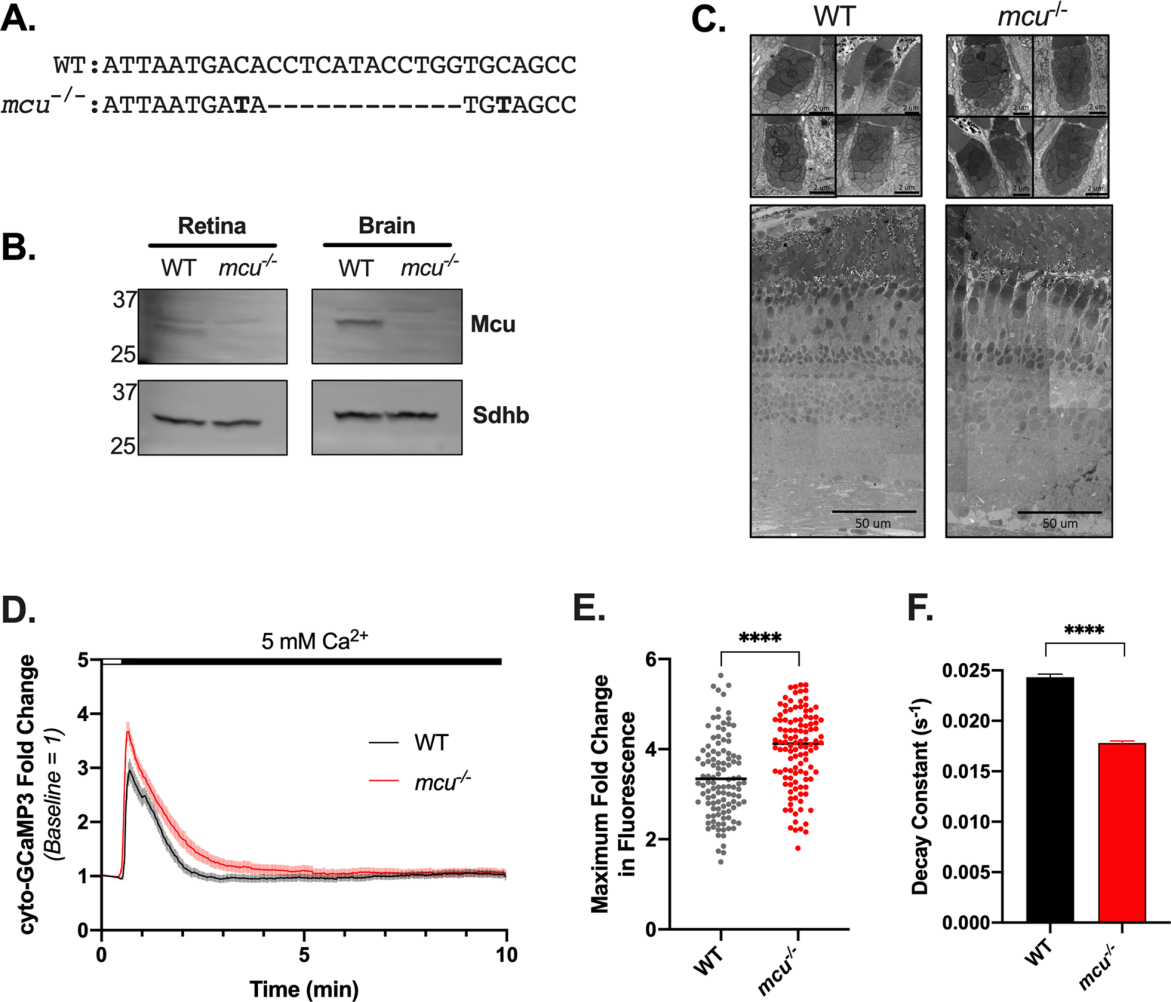
Mcu contributes to clearance of cytosolic Ca^2+^ in zebrafish cones. (**A**) Alignment of a portion of exon 5 of zebrafish *mcu *showing WT (top) and CRISPR-generated *mcu*^-/-^ (*mcu*^*w249*^; bottom). (**B**) Western blot showing Mcu expression in retina and brain from global *mcu*^-/-^ zebrafish. 20 µg of protein from mitochondrial lysate from 6 pooled retinas and 1 brain was analyzed. The custom Mcu antibody detects a faint non-specific band at a slightly higher molecular weight than Mcu. (**C**) Scanning electron microscopy (SEM) images of WT and *mcu*^-/-^ zebrafish cone mitochondria (top panel) and retinas (bottom panel) from 11-month old sibling fish. Retinal and mitochondrial morphology appear unchanged by loss of Mcu (n = 8 retinas from WT and *mcu*^/-^ zebrafish were examined, representative images from 1 WT and 1 *mcu*^-/-^ retina are shown). (**D**) Traces of relative cyto-GCaMP3 fluorescence of cone cell bodies in adult retinal slices of WT or *mcu*^-/-^ fish expressing *gnat2*:cyto-GCaMP3. Baseline mitochondrial fluorescence was determined in KRB buffer containing 0 mM CaCl_2_ and 0.4 mM EGTA, then a bolus of CaCl_2_ was delivered in order to bring the [Ca^2+^]_free_ to 5 mM. The mean is reported and shaded region = 95% CI. (n = 110 cells (four fish) for WT and n = 112 cells (four fish) for *mcu*^-/-^). (**E**) Maximum fold change in cyto-GCaMP3 fluorescence for each cell body after exposure to 5 mM [Ca^2+^]_free_. WT: 3.345 ± 0.085, *mcu*^*-/-*^: 3.985 ± 0.082, mean ± SEM reported, *p* < 0.0001 using Welch’s *t* test. (n = 110 cells (from four fish) for WT and n = 112 cells (from four fish) for *mcu*^-/-^). (**F**) Decay constants calculated using a single exponential decay fit. WT: 0.02433 s^−1^ (0.02384 to 0.02483), *mcu*^*-/-*^: 0.01781 s^−1^ (0.01743 to 0.01821), decay constant with 95% CI reported, *p* < 0.0001 using Welch’s *t* test.

We first generated global *mcu*^-/-^ zebrafish so that we could take advantage of well-established ex vivo retinal Ca^2+^-imaging tools and techniques to determine the role Mcu plays in modulating cytosolic and mitochondrial Ca^2+^ dynamics in cone photoreceptors^21^. Global *mcu*^*-/-*^ zebrafish were generated using a CRISPR-Cas9 strategy outlined previously^22^. A founder carrying a 12-nucleotide deletion in exon 5, which introduces a premature stop codon in exon 6 of *mcu* was used in this study (*mcu*^*w249*^; notated as *mcu*^*-/-*^, Fig. 1A). We used a custom zebrafish-specific Mcu antibody to probe immunoblots of mitochondria isolated from retinas and brains of WT and global *mcu*^-/-^ zebrafish^12^. Mcu expression is entirely ablated in global *mcu*^-/-^ tissues (Fig. 1B). Similar to the global *Mcu*^-/-^ mouse model, a smaller than expected ratio of homozygous *mcu*^-/-^ zebrafish survive to adulthood from crosses of *mcu*^+/-^ parents (Supplemental Fig. 1A)^15,23^. There are no obvious effects of Mcu-deficiency on retinal or photoreceptor morphology (Fig. 1C).

To determine what role Mcu plays in clearing cytosolic Ca^2+^, we crossed *mcu*^*-/-*^ zebrafish with zebrafish expressing the cytosolic Ca^2+^ sensor GCaMP3 (*gnat2:*GCaMP3)^11^. Retinal slices from *mcu*^*-/-*^* gnat2:*GCaMP3 and wild-type (WT) *gnat2:*GCaMP3 siblings were incubated in Krebs–Ringer Bicarbonate (KRB) buffer containing 0 mM CaCl_2_ and 0.4 mM EGTA for 10 min. Cones were then imaged to establish a baseline fluorescence reading prior to delivery of a 5 mM bolus of CaCl_2_ (Fig. 1D). Cones from global *mcu*^*-/-*^ zebrafish exhibit a higher maximum fold change in cytosolic GCaMP3 fluorescence compared to WT cones (Fig. 1E, 1.192 ± 0.024-fold greater than WT, mean ± SEM reported). A single exponential decay was fit to each curve and the decay constant was calculated to determine if WT and *mcu*^*-/-*^ cones clear cytosolic Ca^2+^ at different rates (Fig. 1F). The rate of decay calculated for *mcu*^*-/-*^ (0.01781 s^−1^) was significantly smaller compared to WT (0.02433 s^−1^), indicating that *mcu*^*-/-*^ cones clear cytosolic Ca^2+^ at a slower rate compared to WT cones. This indicates that Mcu plays a role in mediating mitochondrial uptake of cytosolic Ca^2+^ in zebrafish cones.

### Mitochondrial Ca^2+^ uptake in cones from global *mcu*^-/-^ zebrafish is diminished, but not ablated

**Figure 2:**
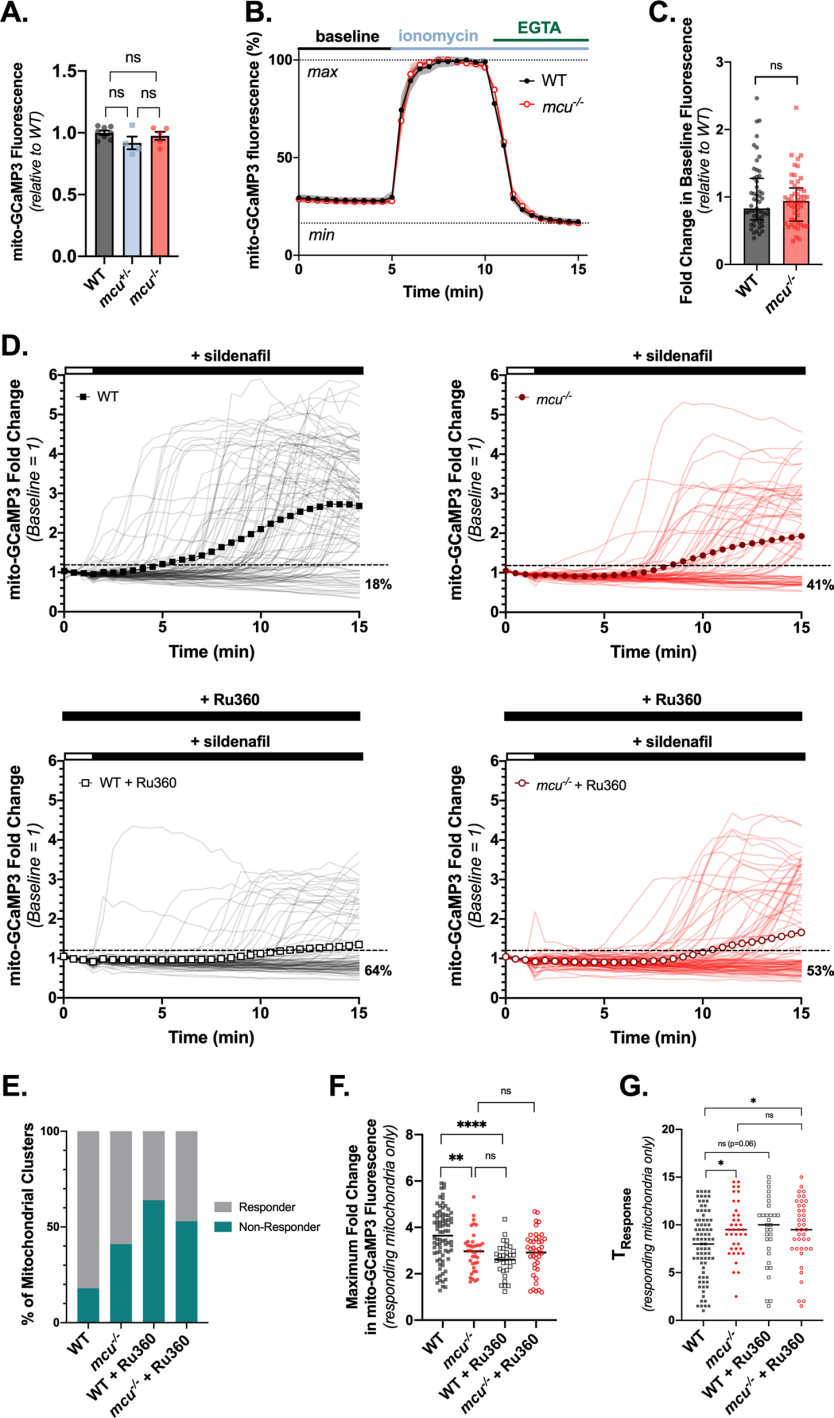
Mitochondrial Ca^2+^ uptake in cones from global *mcu*^-/-^ zebrafish is diminished, but not ablated. (**A**) Total cone mitochondrial fluorescence in *gnat2*:mito-GCaMP3 larval zebrafish eyes. The mean is reported with bars indicating standard error. (n = 8 WT fish, 4 *mcu*^+/-^ fish, and 5 *mcu*^-/-^ fish. ns = not significant using one-way ANOVA with Tukey’s multiple comparisons test). (**B**) Relative mito-GCaMP3 fluorescence of cone mitochondrial clusters in adult retinal slices of WT or *mcu*^-/-^ fish expressing *gnat2*:mito-GCaMP3. Baseline mitochondrial fluorescence was determined in KRB buffer containing 2 mM CaCl_2_, then ionomycin (5 µM) was added to allow 2 mM Ca^2+^ entry into the mitochondria to saturate the probe. Next, EGTA (5 mM) was added to the solution (holding 5 µM ionomycin constant) to chelate Ca^2+^ and determine minimum mito-GCaMP3 fluorescence. The mean is reported and shaded region = 95% CI. (n = 55 mitochondrial clusters (three fish) for WT and n = 51 mitochondrial clusters (three fish) for *mcu*^-/-^). (**C**) Fold change in baseline mito-GCaMP3 fluorescence relative to WT average. The median is reported with bars indicating interquartile range. (ns = not significant using Mann–Whitney test). (**D**) *gnat2*:mito-GCaMP3 retina slices preincubated with 100 µM KB-R7943 (10 min prior to imaging and white bar) then subjected to 25 µM sildenafil (black bar). For Ru360 treatment, retinal slices were preincubated for 1 h in 10 µM Ru360 and then the same experiment was performed in the presence of 10 µM Ru360. The mean response of all mitochondrial clusters is reported with the dark trace, while the semi-transparent traces show the responses of each individual mitochondrial cluster. The dotted line indicates 1.2-fold above baseline. (Mitochondrial clusters from n = 86 WT, n = 64 *mcu*^-/-^, n = 86 WT + Ru360, and n = 85 *mcu*^-/-^ + Ru360 cells are reported. All conditions were tested in multiple slices from n = 3 fish each). (**E**) The percent of mitochondrial clusters from each condition which responded or did not respond to sildenafil. Mitochondrial clusters which exhibited an increase in mito-GCaMP3 fluorescence of 1.2-fold or greater above baseline at any time after sildenafil treatment are considered to have responded. (n = 86 WT, n = 64 *mcu*^-/-^, n = 86 WT + Ru360, and n = 85 *mcu*^-/-^ + Ru360). (**F**) The maximum fold change in mito-GCaMP3 fluorescence at any time during imaging. Mitochondrial clusters which did not respond to sildenafil are excluded. (n = 80 WT, n = 38 *mcu*^-/-^, n = 31 WT + Ru360, n = 40 *mcu*^-/-^ + Ru360). (**G**) The time at which each mitochondrial cluster first increased mito-GCaMP3 fluorescence 1.2-fold above baseline. (n = 80 WT, n = 38 *mcu*^-/-^, n = 31 WT + Ru360, n = 40 *mcu*^-/-^ + Ru360).

To directly observe mitochondrial Ca^2+^ levels in *mcu*^-/-^ photoreceptors, we next crossed *mcu*^-/-^ zebrafish with zebrafish expressing the mitochondrially-targeted Ca^2+^ sensor GCaMP3 in cones (*gnat2*:mito-GCaMP3)^11^. Eyes from live larvae were imaged and we found that there is no difference in basal mito-GCaMP3 fluorescence between WT and *mcu*^-/-^ cones (Fig. 2A). Although we analyzed mito-GCaMP3 fluorescence in sibling larvae, which had only a single insertion of the mito-GCaMP3 transgene, it is possible that differences in transgene expression between fish could obscure differences in WT and *mcu*^-/-^ basal mitochondrial Ca^2+^. So, we next determined basal mitochondrial Ca^2+^ levels in mature cones from WT and *mcu*^-/-^ fish using an ex vivo imaging method that reports mito-GCaMP3 fluorescence independent of mito-GCaMP3 probe concentration. We compared baseline mito-GCaMP3 fluorescence (F_0_) to the maximum fluorescence (F_max_, obtained by addition of ionomycin to media containing 2 mM Ca^2+^) and minimum fluorescence (F_min_, obtained by the addition of 5 mM EGTA to chelate Ca^2+^). Similar to larvae, we found that baseline mito-GCaMP3 fluorescence was not significantly different in adult WT and *mcu*^-/-^ zebrafish cones (Fig. 2B,C).

We next tested whether mitochondrial Ca^2+^ uptake is altered in cones from global *mcu*^-/-^ fish. It has been previously established that the treatment of photoreceptors with the PDE inhibitor sildenafil in the presence of the Na^+^/Ca^2+^ exchanger inhibitor KB-R7943 causes significant increases in cytoplasmic Ca^2+^ levels and a coincident increase in mitochondrial Ca^2+^ levels^11^. We used the same strategy here to assess changes in mitochondrial Ca^2+^ uptake in *mcu*^*-/-*^ cones. We imaged WT and global *mcu*^*-/-*^* gnat2:*mito-GCaMP3 cones after a 10 min KB-R7943 preincubation to obtain baseline measurements of mito-GCaMP3 fluorescence, and continued to image after exposing the retinal slices to sildenafil. In WT cones, mito-GCaMP3 fluorescence increases after sildenafil treatment (Fig. 2D, top left panel). The average response of all WT mitochondrial clusters is shown in the dark trace, and the individual responses of each individual mitochondrial cluster is shown in semi-transparent traces. We determined the number of WT mitochondrial clusters which increase mitochondrial Ca^2+^ levels in response to sildenafil and found that 82% of mitochondrial clusters respond (Fig. 2E; in order for a mitochondrial cluster to have considered to have responded, it must have exhibited a 1.2-fold or greater increase above baseline in mito-GCaMP3 fluorescence at any time during imaging). The population of mitochondrial clusters which did not respond to sildenafil can be visualized by identifying the traces that do not cross the dotted line on the graph indicating 1.2-fold above baseline.

In *mcu*^-/-^ retinal slices, we found that mito-GCaMP3 fluorescence increases after sildenafil treatment in many cones (Fig. 2D, top right panel). However, we observed that there was a greater proportion of *mcu*^*-/-*^ cone mitochondrial clusters which never respond to sildenafil (Fig. 2E, 41% of *mcu*^*-/-*^ mitochondrial clusters never increase 1.2-fold above baseline compared to 18% of WT mitochondrial clusters). The *mcu*^*-/-*^ cone mitochondrial clusters which do respond to sildenafil treatment exhibited a smaller maximum fold change in mito-GCaMP3 fluorescence compared to WT mitochondrial clusters which respond to sildenafil (Fig. 2F). We also observed that *mcu*^*-/-*^ cone mitochondrial clusters take longer to respond to sildenafil treatment compared to WT mitochondrial clusters (Fig. 2G, T_Response_ is defined as the time mito-GCaMP3 fluorescence first increased 1.2-fold above baseline). There is heterogeneity in the timing of the mito-GCaMP3 fluorescence increase from within seconds to many minutes after sildenafil addition in both WT and *mcu*^*-/-*^ cone mitochondrial clusters. However, since *mcu*^*-/-*^ mitochondrial clusters respond later on average, this suggests that a different biological threshold (such as the size of the cytosolic Ca^2+^ load or the amount of time it is sustained) must be met in order for an *mcu*^*-/-*^ mitochondria to begin taking up Ca^2+^.

To assess whether the residual Ca^2+^ uptake exhibited by *mcu*^*-/-*^ cone mitochondrial clusters in this assay could be attributed to an Mcu-independent mechanism, we repeated this experiment in the presence of the Mcu inhibitor Ru360 (Fig. 2D, bottom two panels). Ru360 treatment increases the proportion of WT mitochondrial clusters which never respond (Fig. 2E, 64% of WT + Ru360 mitochondrial clusters do not respond vs 18% of WT mitochondrial clusters without Ru360). WT + Ru360 cone mitochondrial clusters also exhibit a significantly smaller maximum fold change in fluorescence compared to WT mitochondrial clusters without Ru360 (Fig. 2F). Ru360 treatment also slightly increases the proportion of *mcu*^*-/-*^ mitochondrial clusters which never respond (Fig. 2E, 53% of *mcu*^*-/-*^ + Ru360 mitochondrial clusters fail to respond versus 41% of *mcu*^*-/-*^ mitochondrial clusters without Ru360). Compared to untreated *mcu*^*-/-*^ mitochondrial clusters, *mcu*^*-/-*^ + Ru360 mitochondrial clusters do not have a significantly different maximum fold change in fluorescence or T_Response_ (Fig. 2F,G). Notably, a large number of both WT + Ru360 and *mcu*^*-/-*^ + Ru360 mitochondrial clusters still exhibit an apparent increase in mitochondrial Ca^2+^ in this assay.

Since GCaMP3 is a GFP derivative, its fluorescence can be sensitive to changes in pH. The fluorescence of cpYFP (another GFP derivative) is not sensitive to Ca^2+^ but has been shown to be extremely sensitive to changes in pH^24^. To test if the increases in mito-GCaMP3 fluorescence observed in *mcu*^*-/-*^ cone mitochondria and cone mitochondria treated with Ru360 could be due to changes in pH and not mitochondrial Ca^2+^ uptake, we treated retinal slices from *gnat2*:mito-cpYFP zebrafish with KB-R7943 and sildenafil (Supplemental Fig. 2A). We observed no increases in cp-YFP fluorescence over the time course, indicating that the increases in fluorescence we observe with mito-GCaMP3 are due to changes in mitochondrial Ca^2+^ and not pH.

Taken together, these results show that Mcu contributes to some mitochondrial Ca^2+^ uptake in cones, but that Ca^2+^ can also enter cone mitochondria through an alternative pathway.

### Retinas from *mcu*^-/-^ zebrafish have normal morphology, metabolism, and photoresponses

**Figure 3:**
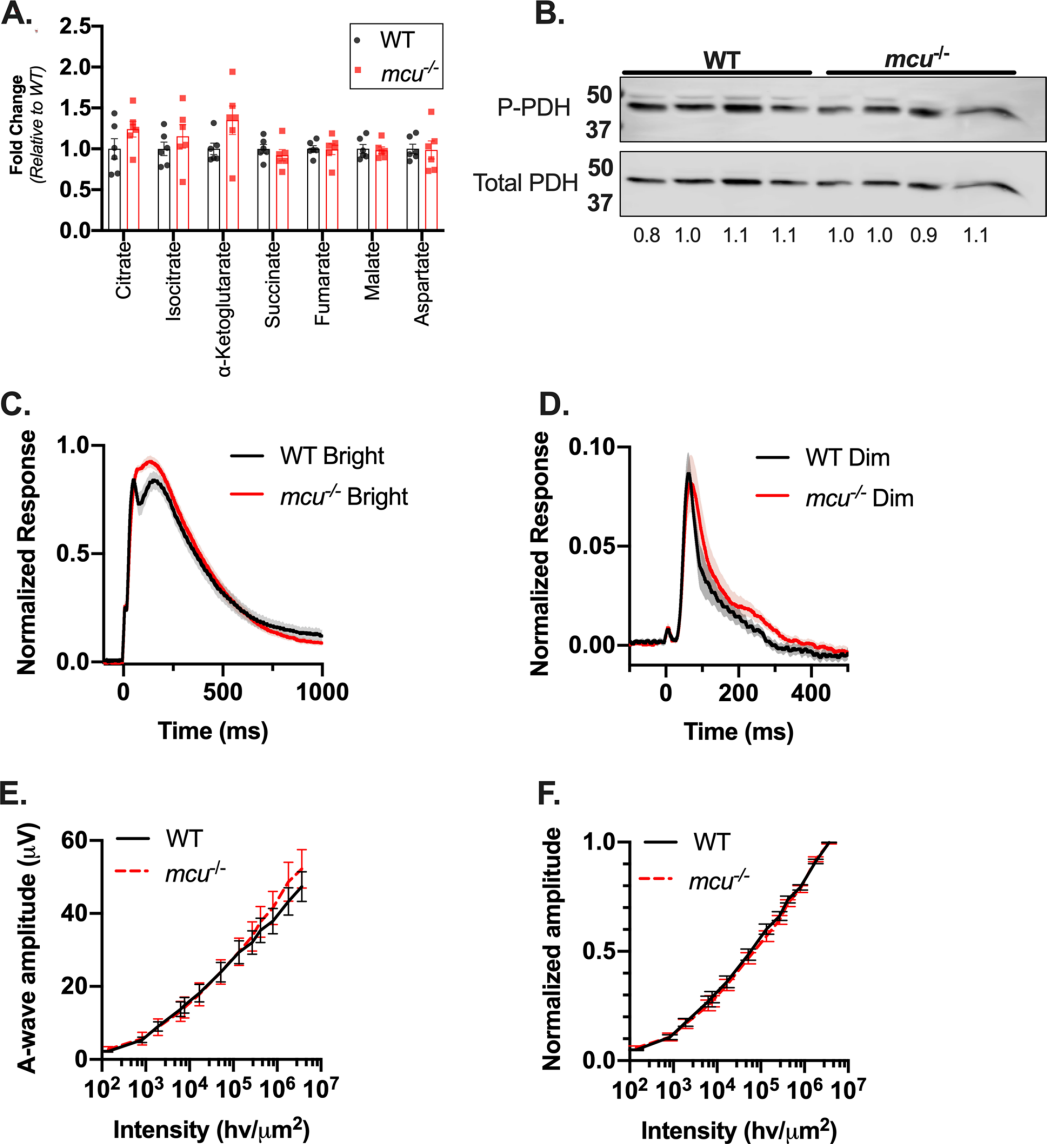
Retinas from global *mcu*^-/-^ zebrafish have normal morphology, metabolism, and photoresponse. (**A**) Total TCA cycle metabolite levels in *mcu*^-/-^ zebrafish retinas relative to WT. Zebrafish were dark-adapted for 18 h and retinas were dissected under red light. α-ketoglutarate levels trend higher in *mcu*^-/-^ zebrafish retinas, although they are not significantly different than WT (1.4 ± 0.5-fold higher in *mcu*^-/-^ retinas, *p* = 0.09 using Welch’s *t* test, mean ± standard deviation is reported, n = 6 WT and 6 *mcu*^-/-^ retinas from 3 different fish each). (**B**) P-Pdh and total Pdh immunoblot from dark-adapted WT and global *mcu*^-/-^ zebrafish retinas. 15 µg of protein was loaded in each lane. Quantification of the P-Pdh/Pdh ratio of each sample relative to the average WT P-Pdh/Pdh ratio is shown below each lane (n = 4 WT and 4 *mcu*^-/-^ retinas from 4 different fish each). (**C**) Ex vivo ERG a-wave responses from WT and *mcu*^-/-^ zebrafish retinas. Cone responses were isolated using DL-AP4 (40 µM) and CNQX (40 µM) and normalized to R_max_ (the maximum response at the brightest light intensity). Bright flash stimulus intensity is 3,650,000 photons µm^−2^ and 2–23 ms in duration. The mean is reported and the shaded region indicates standard error (n = 21 retinas from 12 WT fish and 21 retinas from 13 global *mcu*^-/-^ fish). (**D**) Ex vivo ERG a-wave responses from WT and *mcu*^-/-^ zebrafish retinas under dim flash stimulus under the same conditions as (**C**). (**E**) a-wave response amplitude data plotted as a function of stimulus intensity (photons μm^-2^) of WT and global *mcu*^-/-^ retinas from experiments shown in (**C**) and (**D**) (Bars indicate standard error). (**F**) Normalized response amplitude data for experiments shown in (**C**) and (**D**) indicate that sensitivity is not changed in global *mcu*^-/-^ cones (Bars indicate standard error).

We next determined if the diminished ability of *mcu*^-/-^ mitochondria to take up Ca^2+^ in cones might lead to metabolic or electrophysiological defects. To assess potential changes in metabolism, we measured total metabolite levels from freshly dissected dark-adapted retinas from *mcu*^-/-^ zebrafish. We detected no changes in total metabolite levels in *mcu*^-/-^ retinas, although α-ketoglutarate levels trend slightly but not significantly higher after loss of Mcu (Fig. 3A). Previous studies of MCU^-/-^ tissues describe an increase in the amount of phosphorylated pyruvate dehydrogenase (PDH), which is attributed to diminished activity of the Ca^2+^-sensitive phosphatase PDP1c^15,16,25–27^. We assessed the P-PDH/PDH ratio and found that it is not different between WT and *mcu*^-/-^ retinas (Fig. 3B). This result is consistent with the unaltered resting Ca^2+^ levels we observed in *mcu*^-/-^ cones.

Photoreceptors rely on efficient clearance of cytosolic Ca^2+^ from the outer segment to stimulate recovery of the photoresponse^28^. Photoresponses of zebrafish cones that overexpress Mcu recover faster following a flash of light, suggesting that uptake of Ca^2+^ through Mcu could contribute to clearing outer segment Ca^2+^ in the time course of flash responses^12,29^. Our cyto-GCaMP3 measurements indicate that *mcu*^*-/-*^ cones clear cytoplasmic Ca^2+^ slower compared to WT cones. Thus, loss of this fraction of Mcu-mediated Ca^2+^ sequestration could slow the recovery kinetics of the cone light responses. To test this, we measured both bright and dim flash responses from WT and *mcu*^-/-^ cones using ex vivo electroretinography (ERG). Recovery kinetics are unaltered by Mcu deficiency (Fig. 3C,D). Both the maximum amplitude and sensitivity of the response are unchanged by loss of Mcu expression (Fig. 3E,F, respectively).

### Rod photoreceptors express low levels of MCU

We did not detect any metabolic or physiological consequences caused by Mcu deficiency in zebrafish retinas. However, phenotypes can vary significantly between chronic, induced, and tissue-specific *Mcu*^-/-^ animal models^6^. For example, the cardiac phenotype from the global *Mcu*^-/-^ mouse is surprisingly mild, while constitutive and inducible heart-specific *Mcu* knockdown models have both different and more severe phenotypes^15,18,26,30,31^. This indicates that some tissues have both the capacity and the necessity to adapt to chronic loss of MCU. Since we observed that a smaller than expected number of *mcu*^-/-^ fish reach adulthood from *mcu*^+/-^ crosses, it is likely that the *mcu*^-/-^ fish that survived to adulthood adapted to loss of Mcu in some way. In order to ensure that the mild phenotypes we observed in global *mcu*^-/-^ zebrafish retinas were truly due to the dispensable nature of MCU in photoreceptors and not due to adaptations made in response to chronic loss of MCU, we also generated a rod photoreceptor specific *Mcu*^-/-^ mouse model in which MCU expression is lost only upon photoreceptor differentiation.

**Figure 4:**
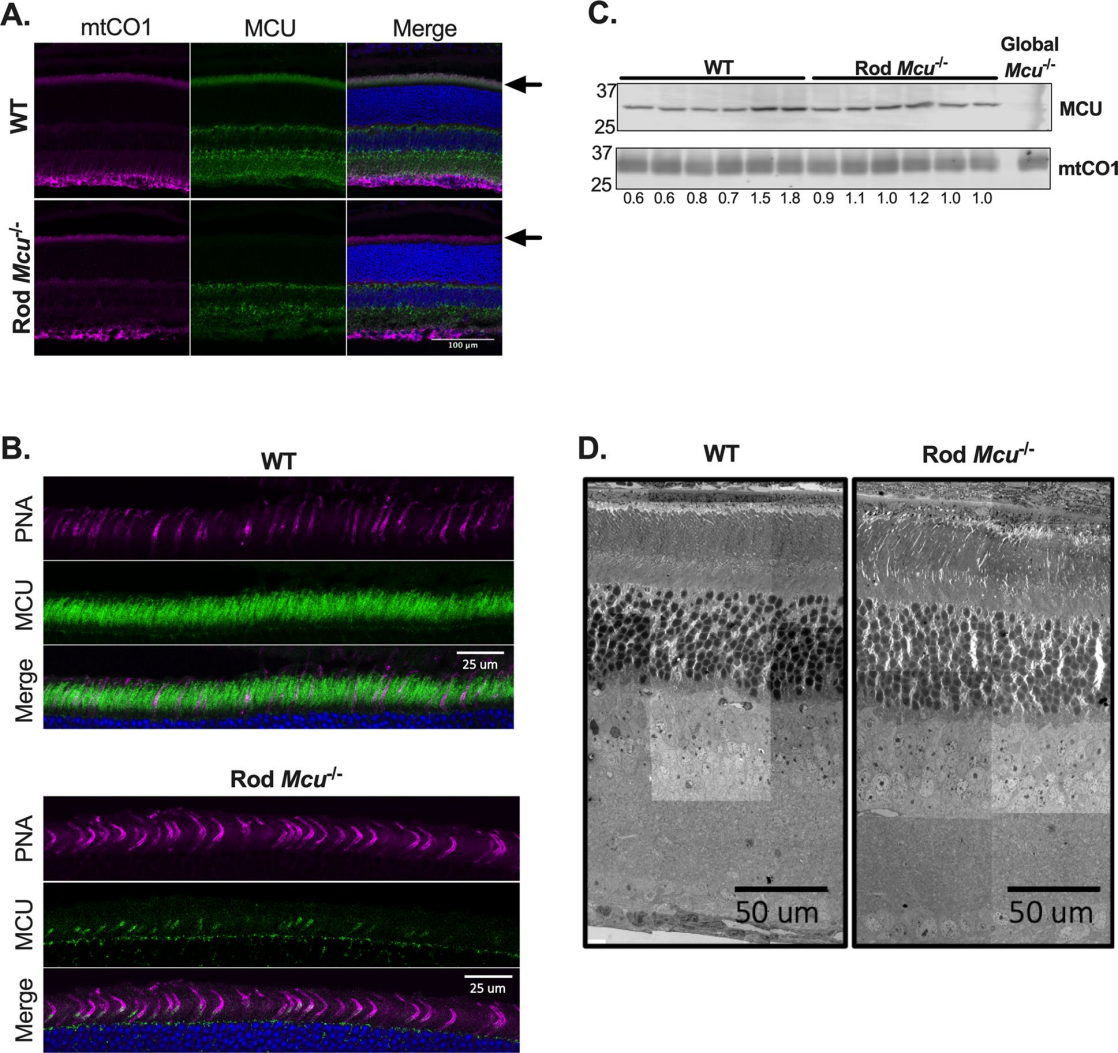
Rods express low levels of MCU. (**A**) Immunohistochemistry showing MCU expression in WT and Rod *Mcu*^*-/-*^ retinas. mtCO1 (Mitochondrial Cytochrome Oxidase subunit 1) is used to label mitochondria. An arrow indicates the photoreceptor mitochondria layer. (**B**) Immunohistochemistry showing MCU expression and PNA-647 (staining cone outer segments). MCU is still expressed in cones from Rod *Mcu*^*-/-*^ retinas. (**C**) Western blot showing MCU expression in whole retinas from Rod *Mcu*^*-/-*^ mouse. 15 µg of retinal protein lysate was loaded in each lane. MCU expression is not significantly altered in Rod *Mcu*^*-/-*^ retinas (1.02 ± 0.03 fold higher in Rod *Mcu*^*-/-*^ retinas, mean ± standard deviation is reported, ns using Welch’s *t* test). Retinal lysate from a global *Mcu*^-/-^ mouse is used as a control to show specificity of MCU antibody (far right lane). The MCU/mtCO1 ratio for each sample relative to WT average is shown under each lane (n = 6 WT and 6 Rod *Mcu*^*-/-*^ retinas from 3 animals each). (**D**) SEM images from 6-month old WT and Rod *Mcu*^*-/-*^ retinas (retinas from n = 3 mice were imaged, representative images from 1 WT and 1 Rod *Mcu*^*-/-*^ retina shown).

We blocked expression of MCU specifically in rod photoreceptors by crossing mice with loxP sites flanking exons 5 and 6 of *Mcu* (*Mcu*^fl^) with mice expressing Cre-recombinase under control of a rod-specific opsin promoter (iCre-75) which is active once rods have differentiated^18,32^. Immunohistochemistry of adult *Mcu*^fl^ iCre-75 (annotated as Rod *Mcu*^-/-^) retinas reveal that MCU expression is ablated in the rod photoreceptors (Fig. 4A, arrow indicates the photoreceptor mitochondria layer). The photoreceptor layer in the mouse retina is composed mainly of rods, with a ~ 35:1 rod:cone ratio^33^. To determine if cones still express MCU in Rod *Mcu*^-/-^ retinas, we co-stained WT and Rod *Mcu*^-/-^ retinal slices with an MCU antibody and the cone marker peanut agglutinin (PNA) (Fig. 4B). We observed MCU staining beneath each PNA-stained outer segment, indicating that cones retain MCU expression.

Cone photoreceptors from zebrafish retinas express very low levels of Mcu^12^. To determine if MCU expression in mouse rods is similarly low, we analyzed MCU expression in WT and Rod *Mcu*^-/-^ retinas using immunoblot (Fig. 4C). MCU expression is not significantly altered in Rod *Mcu*^-/-^ retinas relative to WT, despite rods being by far the most abundant cell type in the mouse retina^33^. This indicates that MCU expression is extremely low in rods relative to other cell types in the retina. Overall retinal and photoreceptor morphology appears to be unaltered by loss of MCU expression in rods: we observed no defects in our immunohistochemistry images and scanning electron microscopy (SEM) analysis of Rod *Mcu*^-/-^ retinas at 6-months old revealed no changes in retinal morphology (Fig. 4D).

### Rod *Mcu*^-/-^ retinas accumulate α-ketoglutarate

**Figure 5:**
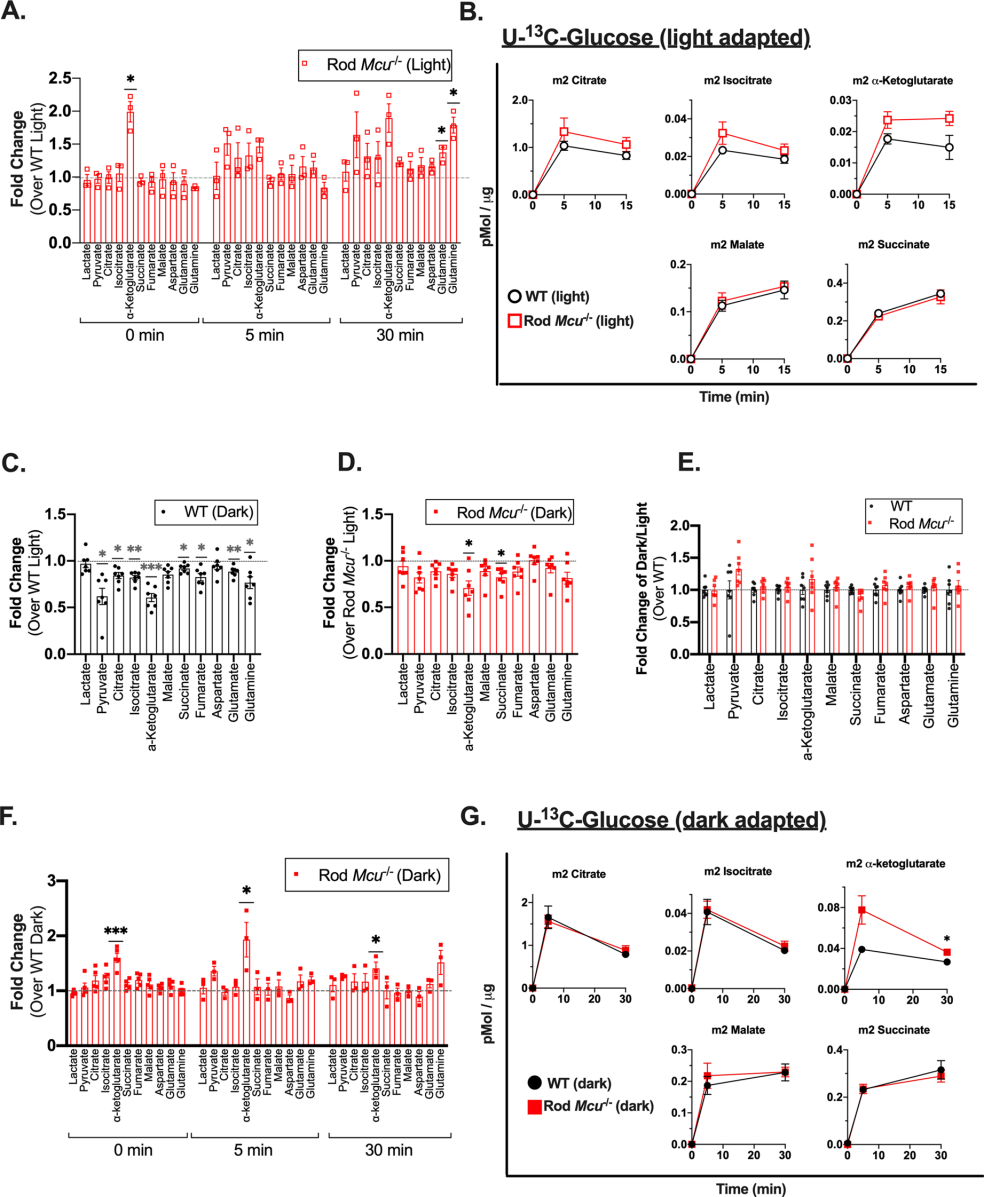
Loss of MCU leads to a buildup of α-ketoglutarate in Rod *Mcu*^*-/-*^ retinas. (**A**) Total metabolite levels in light-adapted Rod *Mcu*^*-/-*^ retinas relative to WT (n = 3 WT and 3 Rod *Mcu*^*-/-*^ retinas per time point. Each time point used retinas from 3 different animals (*indicates *p* < 0.05 using Welch’s *t* test). (**B**) Time course of labeled metabolite accumulation in light-adapted WT and Rod *Mcu*^*-/-*^ retinas incubated in U-^13^C-glucose for 0, 5, and 15 min (n = 3 WT and 3 Rod *Mcu*^*-/-*^ retinas per time point. Each time point used retinas from 3 different mice. (**C**) Total metabolite levels in dark-adapted WT retinas relative to light-adapted WT retinas (n = 4 light adapted retinas and 7 dark adapted retinas, each from four different mice. *indicates *p* < 0.05, **indicates *p* < 0.01, ***indicates *p* < 0.001 using Welch’s *t* test). (**D**) Total metabolite levels in dark-adapted Rod *Mcu*^*-/-*^ retinas relative to light-adapted Rod *Mcu*^*-/-*^ retinas (n = 6 light adapted retinas and 7 dark adapted retinas, each from four different mice. * indicates *p* < 0.05 using Welch’s *t* test). (**E**) Change in metabolite abundance between darkness and light in Rod *Mcu*^*-/-*^ retinas relative to WT retinas from Fig. 4C, D. (n = 4 light adapted WT retinas, 7 dark adapted WT retinas, 6 light-adapted Rod *Mcu*^*-/-*^ retinas, and 7 light-adapted Rod *Mcu*^*-/-*^ retinas, all ns using Welch’s *t* test). (**F**) Total metabolite levels in dark-adapted Rod *Mcu*^*-/-*^ retinas relative to WT (n = 4 WT and 5 Rod *Mcu*^*-/-*^retinas for t = 0, n = 3 WT and 3 Rod *Mcu*^*-/-*^ retinas for t = 5 and t = 30. Each time point used retinas from at least 3 different animals. *indicates *p* < 0.05, *** indicates *p* < 0.001 using Welch’s *t* test). (**G**) Time course of dark-adapted WT and Rod *Mcu*^*-/-*^ retinas incubated in U-^13^C-glucose for 0, 5, and 30 min (n = 3 WT and 3 Rod *Mcu*^*-/-*^ retinas per time point. Each time point used retinas from 3 different animals. *indicates *p* < 0.05 using Welch’s *t* test).

Overexpressing MCU in zebrafish cone photoreceptors leads to changes in the steady-state concentrations of TCA cycle metabolites, likely due to increased Ca^2+^ binding lowering the K_m_ of α-ketoglutarate dehydrogenase (α-KGDH) and isocitrate dehydrogenase^12^. To test if the reduced mitochondrial Ca^2+^ uptake ability of *Mcu*^-/-^ photoreceptors causes a subsequent increase in enzyme K_m_, we used gas chromatography–mass spectrometry (GC–MS) to evaluate the influence of MCU on metabolic flux in retinas. We incubated retinas from WT and Rod *Mcu*^-/-^ light-adapted mice in 5 mM U-^13^C-glucose for 0, 5, and 30 min and quantified accumulation of unlabeled and labeled metabolites using GC–MS (an isotopomer diagram is included in Supplemental Fig. 5B). When we measured total metabolite levels at each time point, we observed that the TCA cycle metabolite α-ketoglutarate was the only metabolite which was consistently elevated in Rod *Mcu*^-/-^ retinas across all time points (α-ketoglutarate levels are elevated 1.99 ± 1.09-fold at 0 min; 1.46 ± 0.36-fold 5 min, and 1.90 ± 0.77-fold at 15 min; mean ± standard deviation propagated to include variation of WT samples reported) (Fig. 5A). Labeled α-ketoglutarate (m2 α-ketoglutarate made in the first round and m3 α-ketoglutarate made in the second round) also accumulate to slightly higher steady state levels in Rod *Mcu*^-/-^ retinas (Fig. 5B, Supplemental Fig. 5A). Other metabolites do not accumulate consistently differently in Rod *Mcu*^-/-^ retinas compared to WT. We also measured the lactate/pyruvate ratio at the 0 min timepoint as a proxy for the cytosolic NADH/NAD^+^ ratio in freshly dissected retinas and found it to be unaltered in Rod *Mcu*^-/-^ retinas (Supplemental Fig. 5C).

Photoreceptor energy demands are significantly higher in darkness, and intracellular Ca^2+^ levels in photoreceptors can increase 10- to 60-fold in darkness relative to light^14,34,35^. So, it is possible that MCU plays a larger role in modulating photoreceptor metabolism in darkness. We first determined how darkness normally alters steady-state metabolite levels by comparing metabolite abundance between light- and dark-adapted WT retinas (for dark-adapted retinas, mice were dark-adapted for 18 h and retinas were dissected and snap-frozen under infrared light). In WT retinas, steady-state levels of many metabolites are slightly but significantly lower in darkness relative to light, with α-ketoglutarate and pyruvate levels being the most reduced (α-ketoglutarate: 0.61 ± 0.12-fold lower; pyruvate: 0.62 ± 0.23-fold lower; mean ± standard deviation propagated to include variation of “light” samples reported) (Fig. 5C). We repeated this comparison using light- and dark-adapted Rod *Mcu*^-/-^ retinas. We observed the same trend in that the steady-state levels of many metabolites are slightly but significantly lower in dark-adapted Rod *Mcu*^-/-^ retinas compared to light-adapted Rod *Mcu*^-/-^ retinas (Fig. 5D). Similar to what was observed in WT retinas, both pyruvate and α-ketoglutarate levels are the most reduced in Rod *Mcu*^-/-^ retinas (α-ketoglutarate: 0.71 ± 0.20-fold lower; pyruvate: 0.82 ± 0.15-fold lower; mean ± standard deviation propagated to include variation of “light” samples reported). When comparing the fold change in metabolite abundance in darkness relative to light, it appeared as though the influence of darkness in Rod *Mcu*^-/-^ retinas tended to be smaller than that of WT retinas. This would be expected if Ca^2+^ uptake via MCU mediated the change in metabolite pool size we observe in darkness relative to light. To test if this were true, we directly compared the fold change in steady-state metabolite levels in darkness and light between WT and Rod *Mcu*^-/-^ retinas (Fig. 5E). While the influence of darkness on pyruvate and α-ketoglutarate is slightly different between WT and Rod *Mcu*^-/-^ retinas, we found that this difference is not statistically significant. This indicates that while MCU might play a small role in modulating steady-state metabolite levels in darkness, it is likely not the primary effector of change.

We next assessed if loss of MCU expression altered metabolic flux in dark-adapted retinas differently than in light-adapted retinas. WT and Rod *Mcu*^-/-^ mice were dark-adapted for 18 h and retinas were dissected, incubated in U-^13^C-glucose, and snap-frozen all under infrared light. Once again, we found that steady-state α-ketoglutarate levels from freshly-dissected dark-adapted retinas are consistently higher in Rod *Mcu*^-/-^ retinas compared to WT (α-ketoglutarate levels are elevated 1.56 ± 0.20-fold at 0 min, 1.93 ± 0.45-fold at 5 min, and 1.41 ± 0.17-fold at 30 min; mean ± standard deviation propagated to include variation of WT samples reported) (Fig. 5F). We also observed a similar increase in Rod *Mcu*^-/-^ m2 and m3 α-ketoglutarate levels throughout the time course of U-^13^C-glucose incubation (Fig. 5G, Supplemental Fig. 5D). We also assessed the levels of lactate, pyruvate, and the lactate/pyruvate ratio (as a proxy for cytosolic NADH/NAD^+^) in freshly dissected and snap-frozen dark-adapted retinas and found it was unchanged in Rod *Mcu*^*-/-*^ retinas (Supplemental Fig. 5E). Overall, we found that metabolic flux in dark-adapted Rod *Mcu*^-/-^ retinas matches flux in dark-adapted WT retinas with the exception of a consistent accumulation of α-ketoglutarate.

Finally, we determined if loss of MCU expression in rods might alter the P-PDH/PDH ratio. Our immunoblot analysis showed that the P-PDH/PDH ratio in light-adapted Rod *Mcu*^-/-^ retinas was not different than in WT (Supplemental Fig. 5F). Accordingly, we did not observe a decrease in citrate production in our isotopic labeling experiments in either light- or dark-adapted Rod *Mcu*^-/-^ retinas.

### MCU-deficiency does not influence mouse rod photoresponses

**Figure 6:**
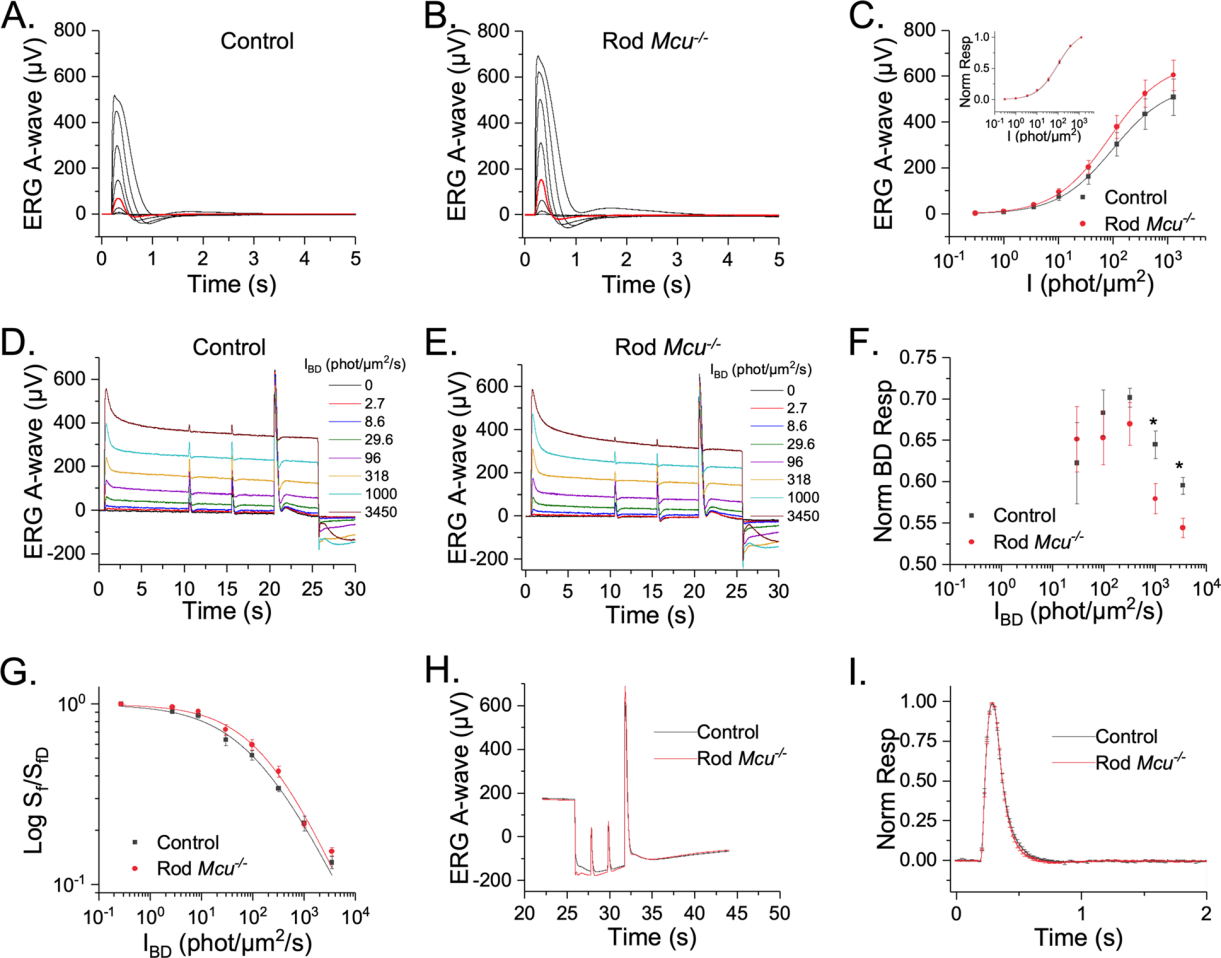
Mouse rods lacking Ca^2+^ uptake through MCU exhibit normal photoresponse. (**A**, **B**) Flash response families of dark adapted *iCre*^+^ (control; **A**) and *Mcu*^*f/f*^* iCre*^+^ (Rod *Mcu*^-/-^; **B**) mice from transretinal ERG recordings. Scotopic a-wave responses were recorded by a series of test flashes (1 ms in duration) with intensities (in photons/µm^2^) 0.3, 1, 3.5, 10.2 (red traces), 35.4, 117, 385, 1270. (**C**) Averaged rod responses (Mean ± SEM) from control (black) and Rod *Mcu*^-/-^ mice (red) plotted as a function of flash intensity show only a marginal (*p* > 0.05) difference in the response amplitude between these groups (n = 10 for each). The solid lines represent curves fitted to the intensity response using the Naka–Rushton Function, R/R_max_ = I/(I + I_1/2_). (Inset) Normalized intensity response curves showing no difference in sensitivity between control (black) and Rod *Mcu*^-/-^ (red) mice. (**D**, **E**) ERG responses to steps of incremental background illumination of control (**D**) and Rod *Mcu*^-/-^ mice (**E**) with subsequent responses to dim and saturating light flashes (n = 8 for each). (**F**) The background light response at plateau, normalized to the peak of the initial background response, as a function of background light intensity. The plateau response for the two brightest backgrounds was significantly lower for the Rod *Mcu*^-/-^ responses compared to these from controls (n = 8 for each). (**G**) Light-adapted sensitivity, normalized to the corresponding dark-adapted value, plotted as a function of background light intensity. Solid lines represent curves fitted to the response plots using the Weber Fechner function. (n = 8 for each). (**H**) Rod sensitivity during subsequent dark adaptation was estimated from ERG responses to dim flashes recorded at 2 s and 4 s after turning off a step of background light; averaged traces for control (black; n = 8) and Rod *Mcu*^-/-^ (red; n = 12) retinas are shown. No notable differences in the kinetics of the dim flash response between control and *Mcu*^*-/-*^ rods at both 2 s and 4 s time points for the two brightest background steps (1000 and 3450 photons/μm^2^/s) were evident. (**I**) A representative plot for the flash responses recorded at 4 s after turning off the highest background (3450 photons/μm^2^/s) shows identical kinetics between control (n = 8) and Rod *Mcu*^-/-^ (n = 12) responses.

We determined how much MCU normally contributes to the rod photoresponse recovery by recording ex vivo transretinal ERG responses from Rod *Mcu*^-/-^ mice and WT controls in scotopic conditions. Dark-adapted flash responses of *Mcu*^-/-^ rods are slightly but not significantly larger than controls (Fig. 6A–C). The normalized flash response family plots superimposed over each other, indicating that the intensity to produce half-maximum response (a measure of sensitivity) of *Mcu*^-/-^ rods is not significantly different from controls (Fig. 6C, *inset*). A summary of the response parameters in the control and *Mcu*^-/-^ rods is given in Table 1. There are no notable differences in the time to peak (T_p_), integration time (T_int_), and recovery time constant τ_rec_ of the flash response.

**Table 1:** Dim flash response sensitivity and kinetics parameters.

	*R*_*max*_ (μV)	*I*_*1*/*2*_ (phot/µm^2^)	*S*_*fD*_ (µV/phot/µm^2^)	*t*_*p*_ (ms)	*t*_*int*_ (ms)	*τ*_*rec*_ (ms)	*I*_o_* (phot/µm^2^/s)	n*
Control (N = 8)	508 ± 79	101 ± 8	0.014 ± 0.001	136 ± 5	359 ± 16	65 ± 4	116 ± 17	0.61 ± 0.06
Rod *Mcu*^-/-^ (N = 8)	605 ± 67	100 ± 18	0.015 ± 0.001	141 ± 6	331 ± 18	65 ± 3	194 ± 37	0.69 ± 0.04
*p* value	0.36	0.94	0.63	0.50	0.28	0.96	0.08	0.3

*R*_*max*_ saturated response amplitude measured at the plateau, *I*_*1/2*_ intensity required to produce half of the saturated response, *S*_*fD*_ dark adapted sensitivity, *t*_*p*_ time to peak of a dim flash response, *t*_*int*_ integration time of the response, *τ*_*rec*_ recovery time constant during response shut off, *I*_*o*_ intensity required to decay the sensitivity to one half.

We next tested if Ca^2+^ sequestering through MCU affects light adaptation of rods. When illuminated, photoreceptors must adapt to the light exposure in order to retain the ability to respond. Ca^2+^ levels in the outer segment of photoreceptors modulate the activity of several proteins to mediate this light adaptation^36–38^. To test if Ca^2+^ sequestering through MCU has an effect on light adaptation of rods, we exposed retinas to a series of background light steps of increasing intensity. The peak response to background light of *Mcu*^-/-^ rods was comparable to controls (Fig. 6D,E). Notably, the background response plateau of *Mcu*^-/-^ rods was lower than the controls and was significant for the two highest background light steps tested (Fig. 6F). However, the light adapted sensitivity (S_f_) remained largely unaffected in Rod *Mcu*^-/-^ compared to the controls across all background light levels tested (Fig. 6G).

MCU activity is controlled by multiple regulatory proteins that confer cooperativity to the channel so that it can robustly respond to changes in cytoplasmic Ca^2+^^39–46^. In the previous experiments, all responses were in either the completely dark-adapted state or in steady-state light adaptation, with cytoplasmic Ca^2+^ being stable at high or low concentrations, respectively. We next examined if MCU modulates responses when photoreceptor intracellular Ca^2+^ levels are rapidly changing and when Ca^2+^ flux through MCU may be changing more dynamically. To do this, we presented test flashes to the retinas that were in the process of dark adapting. After turning off the background light, two test flashes were presented at 2 and 4 s, followed by a saturating light flash (Fig. 6H). This allowed us to assess both flash response kinetics (Fig. 6I) and sensitivity (Table 2). Both parameters were unaltered by the rod MCU deficiency.

**Table 2:** Normalized sensitivity at 2 s and 4 s time points after turning off the background light step.

	*S*_*f*_/*S*_*fD*_ (µV/phot/µm^2^), *I*_*BD*_ (1000 Phot/µm^2^), 2 s	*S*_*f*_/*S*_*fD*_ (µV/phot/µm^2^), *I*_*BD*_ (1000 Phot/µm^2^), 4 s	*S*_*f*_/*S*_*fD*_ (µV/phot/µm^2^), *I*_*BD*_ (3450 Phot/µm^2^), 2 s	*S*_*f*_/*S*_*fD*_ (µV/phot/µm^2^), *I*_*BD*_ (450 Phot/µm^2^), 4 s
Control (N = 8)	1.2 ± 0.1	1.3 ± 0.1	0.96 ± 0.05	1.1 ± 0.04
Rod *Mcu*^-/-^ (N = 12)	1.3 ± 0.1	1.4 ± 0.1	0.9 ± 0.05	1.2 ± 0.06
*p* value	0.46	0.45	0.66	0.73

*S*_*f*_* /S*_*fD*_ normalized fractional sensitivity, *I*_*B*D_ intensity of the background illumination.

### MCU-mediated mitochondrial Ca^2+^ uptake does not contribute to the small photoresponse seen in *Nckx1*^-/-^ mice

The rod Na^+^/Ca^2+^, K^+^ exchanger (NCKX1) is the only known route for Ca^2+^ clearance from the outer segment plasma membrane, and it is thought to be the driver of Ca^2+^ clearance from this compartment. Since MCU expression in mouse rods is extremely low, its contribution to cytosolic Ca^2+^ clearance may be small compared to that of NCKX1. In an effort to unmask any possible modulation of the rod photoresponse by MCU, we investigated the effect of MCU loss on the rod photoresponse in mice lacking NCKX1. NCKX1-deficiency compromises Ca^2+^ extrusion in the rod outer segments and it delays photoresponse recovery following a flash stimulus^29^. If the residual Ca^2+^ extrusion in NCKX1-deficient rods is mediated by MCU, then it would be expected that the subsequent deletion of MCU would further suppress or completely block Ca^2+^ extrusion, causing a further delay in photoresponse recovery and suppressing light adaptation.

**Figure 7:**
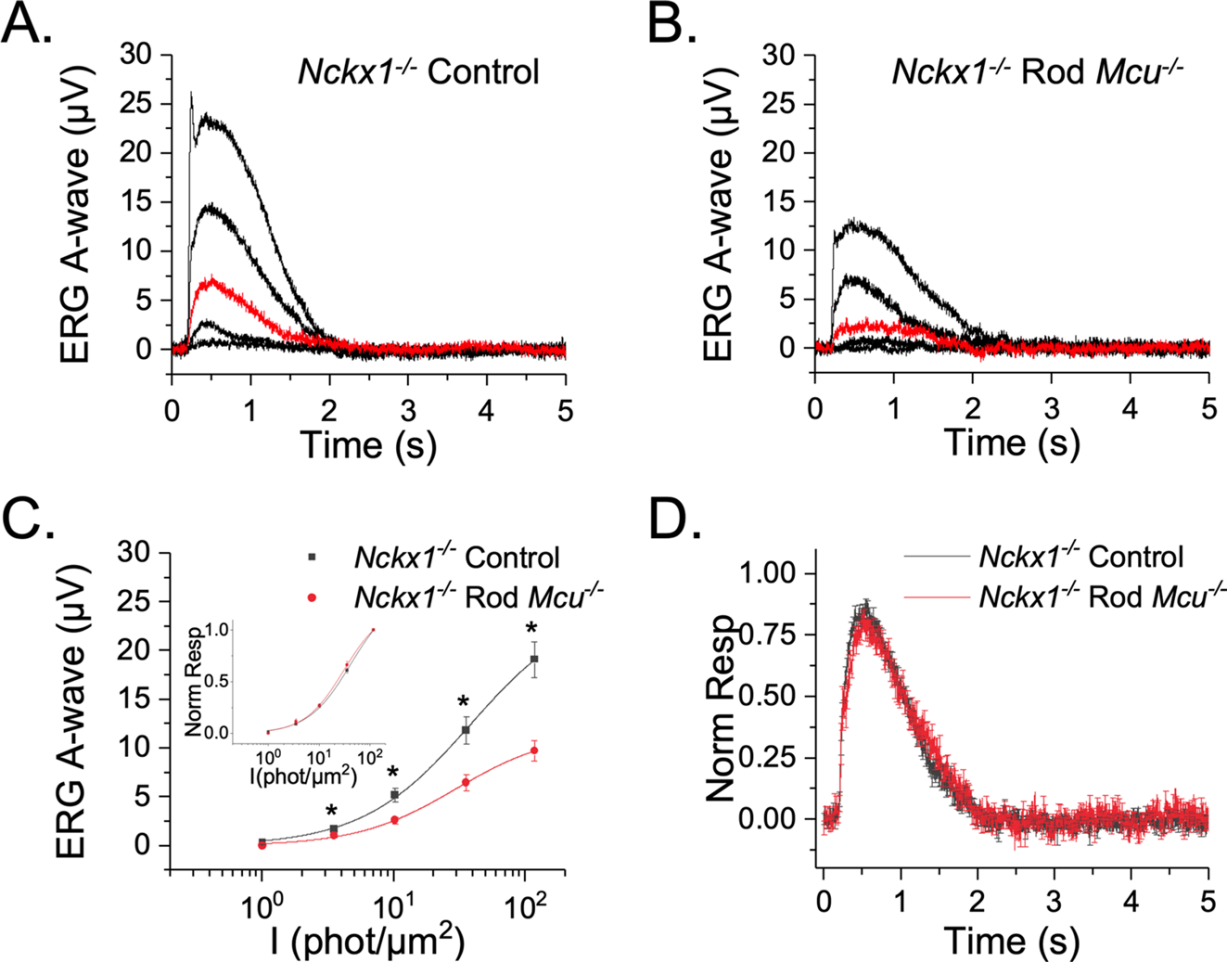
MCU-mediated mitochondrial Ca^2+^ uptake does not modulate the photoresponses in Nckx1^-/-^ mice. (**A**, **B**) Flash response families of dark adapted *Nckx1*^-/-^ iCre^+^ (*Nckx1*^-/-^ control; **A**) and *Nckx1*^-/-^ *Mcu*^f/f^ iCre^+^ double knockout (*Nckx1*^-/-^ Rod *Mcu*^-/-^; **B**) mice from transretinal ERG recordings. Scotopic a-wave responses were recorded by a series of test flashes (1 ms in duration) with intensities (in photons/µm^2^) 1, 3.5, 10.2 (red traces), 35.4, 117. (**C**) Averaged rod responses (Mean ± SEM) from *Nckx1*^-/-^ control (black; n = 10) and *Nckx1*^-/-^ Rod *Mcu*^-/-^ mice (red; n = 9) plotted as a function of flash intensity show a substantial reduction of the response amplitude in the double knockouts as compared to the controls. However, the sensitivity of the rods (estimated their normalized intensity response functions; Inset) remained unchanged between *Nckx1*^-/-^ control (black) and *Nckx1*^-/-^ Rod *Mcu*^-/-^ (red) mice. (**D**) The kinetics of the dim flash response were not affected in the *Nckx1*^-/-^ Rod *Mcu*^-/-^ mice (n = 9); red trace) as compared to *Nckx1*^-/-^ controls (n = 10; black trace).

We recorded transretinal ERG responses from *Nckx1*^-/-^ control and *Nckx1*^-/-^ Rod *Mcu*^-/-^ mice (Fig. 7A,B). As previously shown, deletion of NCKX1 results in a significant reduction in the photoresponse generated by mouse rods (compare Fig. 7A with Fig. 6A)^29^. The responses of *Nckx1*^*-/-*^* Mcu*^*-/-*^ double knockout rods are significantly smaller compared to *Nckx1*^*-/-*^ controls (Fig. 7C). However, the sensitivity and kinetics of the flash response are comparable in *Nckx1*^*-/-*^* Mcu*^*-/-*^ rods and control *Nckx1*^*-/-*^ rods (Fig. 7C inset and D, respectively). This indicates that Ca^2+^ sequestration by MCU does not modulate the rod photoresponse recovery even in the absence of the dominant NCKX1 Ca^2+^ extrusion mechanism. We wondered if the smaller responses of the *Nckx1*^*-/-*^* Mcu*^*-/-*^ double knockout rods might be due to accelerated retinal degeneration, since fewer rods would explain the observed reduction in the photoresponse. However, when we measured the thickness of the different retinal layers of hematoxylin and eosin stained *Nckx1*^-/-^ control and *Nckx1*^*-/-*^* Mcu*^*-/*^- eyes we found no differences, indicating that early retinal degeneration is not responsible for the decreased response (Supplemental Fig. 7A). Table 3 summarizes the rod response properties of *Nckx1*^*-/-*^ controls and *Nckx1*^*-/-*^ Rod *Mcu*^*-/-*^ mice. Overall, these results indicate that Ca^2+^ uptake mediated by MCU does not significantly influence the rod photoresponse.

**Table 3:** Dim flash response sensitivity and kinetics parameters.

	*R*_*max*_ (μV)	*I*_1/2_ (phot/µm^2^)	*S*_*fD*_ (µV/phot/µm^2^)	*t*_*p*_ (ms)	*T*_*int*_ (ms)	*τ*_*rec*_ (ms)
Nckx1^-/-^ Control (N = 10)	19 ± 2	22.8 ± 1.4	0.02605 ± 0.001	312.9 ± 19	1564 ± 83	441.3 ± 52
Nckx1^-/-^ Rod *Mcu*^-/-^ (N = 9)	9.7 ± 1	23.2 ± 2	0.02555 ± 0.002	367.7 ± 24	1687 ± 81	501 ± 103
*p* value	0.0005	0.87	0.86	0.09	0.3	0.6

*R*_*max*_ saturated response amplitude measured at the plateau, *I*_*1/2*_ intensity required to produce half of the saturated response, *S*_*fD*_ dark adapted sensitivity, *t*_*p*_ time to peak of a dim flash response, *t*_*int*_ integration time of the response, *τ*_*rec*_ recovery time constant during response shut off.

## Discussion

Photoreceptors have abundant mitochondria in the compartment of the cell between the nucleus and the outer segment that can influence cytosolic Ca^2+^ pools^11^. Photoreceptors also depend on Ca^2+^ homeostasis for proper function and viability^9,10^. Despite this, we find that loss of the presumptive primary Ca^2+^ channel for mitochondrial Ca^2+^ uptake (MCU) is tolerated surprisingly well by photoreceptors. MCU-deficient photoreceptors appear healthy, their TCA cycle activity is largely unaltered, and the photoresponse is preserved. In cones from global *mcu*^-/-^ zebrafish, cytosolic Ca^2+^ is cleared more slowly and mitochondrial Ca^2+^ uptake is reduced in a significant population of mitochondria. However, some mitochondria still display robust increases in mito-GCaMP3 fluorescence in the absence of MCU. Ca^2+^ uptake in these mitochondria appears to occur more slowly and with a lower magnitude than in WT mitochondria. Consistent with this result, we found that both WT and *mcu*^-/-^ cones have a population of mitochondrial clusters which still appear to take up Ca^2+^ even in the presence of the MCU inhibitor Ru360, and the characteristics of this population match those of the responding mitochondria in *mcu*^*-/-*^ cones. This is similar to what has been observed in non-synaptic brain mitochondria isolated from *Mcu*^*-/-*^ mice, in which uptake of Ca^2+^ into mitochondria was not blocked but instead occurred at a slower rate compared to controls^20^. Taken together, these results suggest that that neuronal tissue like retina and brain may have an alternative mitochondrial Ca^2+^ uptake pathway with different response kinetics and Ca^2+^ uptake capacity. Future experiments which use other strategies for measuring mitochondrial Ca^2+^ uptake will be critical for confirming, identifying, and assessing the role of MCU-independent mechanisms for mitochondrial Ca^2+^ uptake in photoreceptors.

Other candidates for Ca^2+^ entry have been described but are not as well-characterized as MCU, as MCU is thought to be primarily responsible for mitochondrial Ca^2+^ influx. However, the viability *of Mcu*^*-/-*^ mice and zebrafish, and the extremely mild consequences of MCU loss in photoreceptors we describe here, suggest that there is an alternative uptake mechanism in at least some cell types^15,47^. Letm1 is a Ca^2+^/H^+^ exchanger on the inner mitochondrial membrane that links Ca^2+^ influx and efflux to electron transport chain activity and mitochondrial pH^48^. Letm1 appears to act primarily as a Ca^2+^ extrusion mechanism, but it is also capable of Ca^2+^ influx. Knockdown of Letm1 impairs Ca^2+^ transport into the mitochondria of Flp-In-293 cells and patient-derived fibroblasts, and Letm1^+/-^ mice have impaired ATP production and PDH activity specifically in neuronal tissue^49,50^. There is also some evidence that mitochondrial ryanodine receptors may exist in neurons and cardiac cells, but they have yet to be unambiguously identified^51,52^. Another candidate is the mitochondrial Na^+^/Ca^2+^ exchanger, which is associated with Ca^2+^ efflux but can run in reverse to promote Ca^2+^ entry into mitochondria^53^.

A possible advantage of relying on these other mechanisms for Ca^2+^ transport is that it would link mitochondrial Ca^2+^ influx to factors other than cytosolic Ca^2+^ levels. The mitochondrial Na^+^/Ca^2+^ exchanger would couple Ca^2+^ entry to cytosolic Na^+^, which fluctuates in photoreceptors in response to light. Letm1 would tie Ca^2+^ entry to mitochondrial pH and ATP production, and Letm1 has even been shown to be upregulated in cancer tissue, which is highly glycolytic like photoreceptors^54^. Whatever the alternative pathway of mitochondrial Ca^2+^ uptake might be in photoreceptors, the low expression of MCU in photoreceptors raises the possibility that this alternative pathway could play a significant role in controlling photoreceptor mitochondrial Ca^2+^ uptake.

Loss of MCU has very mild metabolic consequences for photoreceptors. Evidence continues to suggest that tissue-specific metabolic specialization causes the metabolic consequences of MCU loss to vary from to tissue. For example, the liver has the important role of synthesizing fatty acids for the body and can also store lipids. In mouse and zebrafish hepatocytes, loss of MCU disrupts lipid metabolism and leads to hepatic lipid accumulation due to delayed cytosolic Ca^2+^ clearance and subsequent disrupted AMP-activated protein kinase dephosphorylation^47^. Conversely, in skeletal muscle, loss of MCU causes a metabolic shift towards increased lipid oxidation due to PDH inhibition restricting the availability of pyruvate-derived acetyl-CoA^17,27^. These examples suggest that the basal metabolic specialization of a tissue will determine in large part the metabolic consequences of MCU loss. Since photoreceptors rely heavily on aerobic glycolysis to generate ATP, it is not entirely surprising that Rod *Mcu*^*-/-*^ mouse retinas are metabolically quite normal and exhibit only a mild accumulation of α-ketoglutarate that does not appear to affect photoreceptor function (Fig. 5).

α-ketoglutarate participates in multiple mitochondrial and cytosolic reactions, making it difficult to pinpoint the exact reaction that is altered by loss of MCU in rods which leads to this accumulation. This is not the first time an accumulation of α-ketoglutarate has been observed to accompany MCU loss, as a similar accumulation of α-ketoglutarate occurs in *Mcu*^*-/-*^ fibroblasts. These fibroblasts have defects in glucose metabolism upstream of α-ketoglutarate, and the accumulated α-ketoglutarate was attributed to upregulated glutaminolysis^55^. However, *Mcu*^*-/-*^ photoreceptors exhibit no defects in glucose metabolism upstream of α-ketoglutarate and we see no evidence for altered synthesis of glutamate or glutamine in retinas supplied with U-^13^C-glucose, which suggests a different mechanism is responsible.

An alternative possibility is that α-ketoglutarate levels increase after loss of MCU due to decreased Ca^2+^ stimulation of α-KGDH. When α-KGDH is bound to Ca^2+^, its K_m_ for α-ketoglutarate is lowered^3,56^. We previously observed that increased matrix Ca^2+^ in zebrafish cone photoreceptors lowers the steady-state concentration of α-ketoglutarate, presumably because less substrate is needed to activate α-KGDH due to its lowered K_m_^12^. Since steady-state α-ketoglutarate levels increase in MCU-deficient retinas, it is reasonable to hypothesize that the opposite occurs: more substrate is needed to activate α-KGDH due to its increased K_m_ when one route of Ca^2+^ entry into the matrix (MCU) is shut down (Fig. 5). However, our measurements of matrix Ca^2+^ show that the overall basal matrix Ca^2+^ levels are unaltered in cone mitochondria lacking MCU (Fig. 2). We suggest the following possible explanations for elevated α-ketoglutarate levels in the absence of a detectable change in matrix Ca^2+^:

1. Matrix Ca^2+^ levels are decreased in rods lacking MCU but not in cones from global *mcu*^-/-^ zebrafish. Genetically encoded, mitochondrially-targeted Ca^2+^-sensors with rod-specific expression do not exist in mice, and we are unable to selectively purify rod mitochondria from the rest of the inner retinal mitochondria, so we were unable to directly measure matrix Ca^2+^ levels in rod photoreceptors.
2. Loss of MCU results in subtle changes in matrix Ca^2+^ microdomains which influence α-KGDH activity but that we are unable to detect using our imaging methods
3. α-ketoglutarate levels are affected by an unknown, matrix Ca^2+^-independent process (such as altered malate-aspartate shuttle activity or changes in another metabolic reaction involving α-ketoglutarate)

*Mcu*^*-/-*^ rods exhibit a normal photoresponse with no change in the sensitivity and kinetics of the flash response and light adaptation (Fig. 6). However, mitochondrial Ca^2+^ uptake via MCU is regulated cooperatively by several regulatory proteins^39–42^. So, we also investigated if MCU might play a role in modulating the photoresponse only when intracellular Ca^2+^ levels are more rapidly changing, such as when photoreceptors are adapting to background light or during the subsequent recovery back to their dark adapted state. However, *Mcu*^*-/-*^ rods did not display a change in photoresponse parameters even while dark adapting following exposure to background light. This lack of phenotype may reflect the extremely low expression of MCU in mouse rods relative to the more dominant Ca^2+^ clearance pathways such as NCKX1.

To determine if MCU might play a small role in clearing Ca^2+^ that is difficult to detect in the presence of other Ca^2+^ clearance pathways, we compared photoresponses of single knockout *Nckx1*^*-/-*^ mice and double knockout *Nckx1*^*-/-*^ Rod *Mcu*^*-*/-^ mice. *Nckx1*^*-*/-^ mice maintain a small photoresponse and are remarkably slow to degenerate, which indicates that they are able to clear a small amount of outer segment Ca^2+^ through a not yet understood pathway^29^. We hypothesized that if the small photoresponse observed in *Nckx1*^*-/-*^ mice were due to Ca^2+^ sequestering via MCU, that this response would be ablated in the double knockout *Nckx1*^*-/-*^ Rod *Mcu*^*-/-*^ mice. However, the sensitivity, flash response kinetics and even light adaptation were unchanged in *Nckx1*^*-/-*^ Rod *Mcu*^*-/-*^ mice compared to *Nckx1*^*-/-*^ controls, which indicates that mitochondrial Ca^2+^ uptake via MCU does not contribute substantially to outer segment Ca^2+^ clearance or photoresponse recovery (Fig. 7). It remains a possibility that mitochondrial Ca^2+^ uptake through an alternative pathway contributes to the small photoresponse that is seen in *Nckx1*^*-/-*^ Rod *Mcu*^*-/-*^ mice. We did observe a reduction in the flash response amplitude in *Nckx1*^*-/-*^ Rod *Mcu*^*-/-*^ as compared to *Nckx1*^*-/-*^ rods. The reason for this remains unclear. Although we confirmed that there was no obvious degeneration of rods, it is still possible that there is a subtle shortening of the rod outer segments that we were unable to detect in our sections. Overall, we find that MCU does not play a significant role in Ca^2+^ feedback mechanisms during the photoresponse.

Maintaining intracellular Ca^2+^ homeostasis is vital for many cell types, including photoreceptors. While most cells are thought to facilitate mitochondrial Ca^2+^ uptake primarily through MCU, we find that both rod and cone photoreceptors have limited MCU expression. In the absence of MCU, cone mitochondrial Ca^2+^ uptake is diminished but not entirely ablated. We found that this fraction of MCU-mediated mitochondrial Ca^2+^ uptake has a very limited effect on modulating metabolism and no role in modulating the photoresponse. However, this does not mean that mitochondria do not play a role in modulating photoreceptor function by buffering intracellular Ca^2+^, as we find evidence for MCU-independent mitochondrial Ca^2^^+^ uptake in photoreceptors. Overall, we find that MCU is surprisingly dispensable for photoreceptor function, possibly in favor of an alternative mitochondrial Ca^2+^ uptake pathway.

## Methods

### Animal use

Mice were maintained and used in accordance with the guidelines of experimental protocols approved by the Institutional Animal Care and Use Committees (IACUC) of Washington University in St. Louis (protocol approval number: 20170232) and the University of Washington in Seattle (protocol number: 2050-01). Zebrafish were maintained and used in accordance with the guidelines of experimental protocols approved by the IACUC of both the University of Washington in Seattle (protocol number: 3113-02) and University of Utah (protocol number: 17-11004).

### Animal care

At Washington University in St. Louis, mice were kept under a 12 h light/dark cycle and given free access to food and water. At the University of Washington, mice were housed in the UW Medicine SLU 3.1 vivarium, where they experienced a 6 AM–9 PM (fall-winter) and 7 AM–9 PM (spring–summer) light/dark cycle and had free access to food and water. In Figs. 4 and 5, “WT” animals are *Mcu*^*fl/fl*^ iCre-negative littermates. In Fig. 6, “Control” animals are iCre-positive  mice in which *Mcu* is not floxed. In Fig. 7, “Control” animals are *Nckx1*^-/-^ single knockouts. In Figs. 5 and 6, the control and experimental mice were obtained from separate lines derived from common parents. iCre-75 mice were a gift from the lab of Ching Kang (Jason) Chen^32^. MCU^fl^ mice (B6;129S-*Mcu*^*tm1.1Jmol*^/J) were obtained from Jackson Labs. The *Nckx1*^-/-^ mice used in this study were generated as described in a previous publication^29^. Mice were maintained on a C57BL/6 J background. Rod *Mcu*^*-/-*^ mice used for histology were 6 and 11 months of age (6 months of age is shown in Fig. 1), mice used for metabolic analysis were between 5 and 9 months of age, mice used for ERG analysis were 6 to 8 weeks of age, and *Nckx1*^*-/-*^ and *Nckx1*^*-/-*^ Rod *Mcu*^*-/-*^ mice used for histology were 6 to 8 weeks of age. Mice were genotyped to confirm the absence of the Rd8 mutation. Equal numbers of male and female mice were used in this study.

All fish used in this analysis were maintained in the University of Washington South Lake Union aquatics facility or the Centralized Zebrafish Animal Resource (CZAR) at the University of Utah at 27.5 °C on a 14/10 h light/dark cycle and were maintained in the Roy^-/-^ genetic background. All wild-type fish (WT) used in analysis were age-matched siblings to CRISPR-generated *mcu*^*-/-*^ zebrafish. Fish used for mito-GCaMP3 slice preparation were between 7 and 11 months of age, fish used for cyto-GCaMP3 slice preparation were 18 months of age, fish used for metabolic analysis were 11 months of age, fish used in ERG analysis were 7 months of age, and fish used for retinal morphology analysis were 11 months of age. Equal numbers of male and female fish were used.

### Immunoblotting

Protein was extracted by homogenizing in RIPA buffer (150 mM NaCl, 1.0% Triton X-100, 0.5% sodium deoxycholate, 0.1% SDS, 50 mM Tris, pH 8.0) and run on 14% polyacrylamide gels. After running, gels were transferred onto PVDF membranes (Millipore, IPFL00010) and briefly washed with PBS. Primary antibodies were diluted in blocking buffer (LI-COR, 927–40,000) and incubated overnight on blots at 4 °C. Membranes were washed twice with PBS containing 0.1% Tween-20 and once with PBS, then incubated with secondary antibodies diluted 1:5000 in blocking buffer for 1 h at RT and washed again before imaging. Membranes were imaged and bands were quantified using the LI-COR Odyssey CLx Imaging System (RRID:SCR_014579). Primary antibodies used: PDH E1 subunit (Abcam Cat# ab110334, RRID:AB_10866116), P-PDH (EMD Millipore Cat# ABS204, RRID:AB_11205754), mtCO1 (Abcam Cat# ab14705, RRID:AB_2084810), SDHB (Abcam Cat# ab14714, RRID:AB_301432), and Cell Signaling MCU (Cell Signaling Technology Cat# 14997, RRID:AB_2721812), custom MCU antibody^12^. All primary antibodies were used at 1:1000, with the exception of the custom MCU antibody which was used at 1:100. Secondary antibodies used: IRDye 800CW donkey anti-rabbit IgG (H + L) (LI-COR Biosciences, 925-32213, RRID: AB_2715510); IRDye 680RD donkey anti-mouse IgG (H + L) (LI-COR Biosciences, 925-32212, RRID: AB_2716622); IRDye 680RD donkey anti-rabbit IgG (H + L) (LI-COR Biosciences, 925-68073, RRID: AB_2716687); IRDye 800CW goat anti-mouse IgG (H + L) (LI-COR Biosciences, 925-32210, RRID: AB_2687825).

### Immunohistochemistry

For best immunohistochemistry results, mice were perfused with fixative according published methods^57^. Briefly, PBS was placed in a 37 °C water bath and all fixative tubing was flushed repeatedly with PBS to clear any bubbles. Mice were anaesthetized using 270 mg/kg of nebutol. Mice were taped to a dissection board and open-heart surgery was performed in order to insert the perfusion needle into the left ventricle. The right atrium was snipped, and PBS was perfused through the mouse using a peristaltic pump until the outflow was clear. Then, room temperature 4% PFA (prepared from 16% PFA diluted in PBS) was perfused through the mouse. After fixation, eyes were carefully cut out with curved dissection scissors, the sclera was cut off, and eyes were stored in 4% PFA for 2 h. Eyes were then rinsed with PBS, and moved through a sucrose gradient of 5%, 10%, 20%, and 30% sucrose (eyes were transferred to increasing concentrations of sucrose each time they sank to the bottom of the tube). Eyes were embedded in O.C.T. compound and 20 micron sections were cut using a Leica cryostat. For immunostaining, sections were rehydrated with PBS for 10 min and blocked in Normal Goat Serum (NGS) for 1 h. Primary antibodies were diluted in NGS and incubated on sections overnight at 4 °C in a humidified chamber. Sections were washed three times with PBS, and secondary antibodies were diluted in PBS and incubated on sections for 1 h at room temperature. Sections were washed 3 × with PBS, with the middle wash containing 5 μM Hoechst nuclear stain diluted in PBS (Hoechst 33342, Trihydrochloride, Trihydrate stain (ThermoFischer, H3570). Slides were mounted using Fluoromount (Southern Biotech Cat#: 0100-01) and imaged using an Olympus FV1000 Confocal microscope. Antibodies and stains used were: mtCO1 1:500 (Abcam Cat# ab14705, RRID:AB_2084810); MCU 1:2000 (Cell Signaling Technology Cat# 14997, RRID:AB_2721812); PNA 1:200 (after suspending at a concentration of 1 mg/mL in H20) (Lectin PNA Alexa Fluor 647 conjugate, ThermoFischer Cat# L32460); Goat anti-Rabbit IgG (H + L) Alexa Fluor 633 (Thermo Fisher Scientific, Cat# A-21070, RRID AB_2535731; Goat Anti-Mouse IgG H&L Alexa Fluor 488 (Abcam, ab150113, RRID:AB_2576208).

### Isotopic labeling

Krebs–Ringer bicarbonate (KRB) buffer (98.5 mM NaCl, 4.9 mM KCl, 1.2 mM KH_2_PO_4_ 1.2 mM MGSO_4_–7H_2_O, 20 mM HEPES, 2.6 mM CaCl–2H_2_O, 25.9 mM NaHCO_3_) optimized for isotopic labeling experiments in retinas was used in these experiments. Mice were euthanized by awake cervical dislocation and eyes were rapidly enucleated into a dish of Hank’s Buffered Salt Solution (HBSS; Gibco, Cat#: 14025-076). For flux measurements, retinas were placed in pre-warmed KRB containing d-[U-^13^C]-glucose (Cambridge Isotope Laboratories, CLM-1396). Retinas were incubated for the specified time points at 37 °C mouse at 5% CO_2_ and room oxygen, then washed twice in ice-cold PBS and flash frozen in liquid nitrogen. For mouse dark-adapted experiments, mice were dark-adapted for a minimum of 18 h. Samples were collected exactly as above, but in complete darkness under an infrared light using night vision goggles. For zebrafish dark-adapted experiments, zebrafish were dark adapted for 18 h, zebrafish were euthanized using an ice bath, and samples were collected and snap-frozen under dim red light.

### Mass spectrometry sample preparation

Metabolites were extracted from retinas using ice-cold 80% MeOH. 150 μL extraction buffer was added to each sample and tissue was disrupted by sonication. Samples were then spun at maximum speed, the supernatant transferred to a new tube, and the pellet saved for protein quantification. The supernatant was lyophilized at room-temp until dry. Extracted metabolites were derivatized using a two-step process: (1) 10 μL of 20 mg/mL Methoxyamine HCl (Sigma, Cat#: 226904) dissolved in pyridine (Sigma, Cat#: 270970) was added and samples were incubated at 37 °C for 90 min, then (2) 10 μL of *tert*-butyldimethylsilyl-*N*-methyltrifluoroacetamide (Sigma, Cat#: 394882) was added and samples were incubated at 70 °C for 90 min. Metabolites were analyzed on an Agilent 7890/5975C GC–MS using selected-ion monitoring methods described in previous work^7–10^. Peaks were manually integrated using MSD ChemStation software (Agilent), and correction for natural isotope abundance was performed using Isocor software^58^. Raw signals for each metabolite were converted to molar amounts using metabolite standard curves which were run alongside each experiment. Molar amounts were normalized to the total amount of protein (determined using a BCA assay) for each sample to determine the molar amount per μg of cellular protein. Statistical analysis and figure preparation was performed with GraphPad v 8.4.3 (for Mac, GraphPad Software, San Diego CA, USA, www.graphpad.com).

### Live larval imaging of mito-GCaMP3

Larvae were imaged as described previously^12^. Larvae were maintained in embryo media containing 0.0003% 1-phenyl 2-thiourea (PTU, Sigma-Aldrich P7629) starting at 20 h postfertilization for confocal imaging. Larvae were analyzed at 6 days postfertilization (dpf) by embedding in 0.5% low melting point agarose containing embryo media with 0.02% (w/v) Tricaine (Sigma-Aldrich, E10521). The agarose was submerged in embryo media containing 0.0003% PTU and 0.02% (w/v) tricaine. Imaging was performed using an Olympus FV1000 with a 40 × water objective in conjunction with Olympus FluoView FV10-ASW software (RRID:SCR_014215). The excitation/emission wavelengths used for mito-GCaMP3 were 488/510 nm. Images of total eye mitochondrial clusters were collected at a z-depth of 2 µm, and blinded quantification was performed using ImageJ + Fiji software (SCR_002285).

### Retinal slice imaging of cyto-GCaMP3 and mito-GCaMP3

Slices were prepared as described previously^11,21^. For cyto-GCaMP3 measurements, retinal slices were incubated in KRB buffer (containing 0 mM CaCl_2_ and 0.4 mM EGTA) for 10 min. For basal cyto-GCaMP3 determination, slices were imaged every 2 s for 24 s to establish baseline cyto-GCaMP3 fluorescence. A bolus of CaCl_2_ was then injected in the imaging chamber to bring the final [Ca^2+^]_free_ in the chamber to 5 mM. Retinal slices were imaged for a total of 10 min to monitor Ca^2+^ clearance. For basal mito-GCaMP3 determination, 15 z-slices of 2 µm thickness were collected every 30 s. Retinas were incubated in KRB buffer (containing 2 mM CaCl_2_) for 5 min, then the chamber was injected with ionomycin to a final concentration of 5 µM (Sigma, 407950, prepared in DMSO) for another 5 min of image collection. An excess of EGTA (5 mM) was then injected and images were collected for another 5 min. For mitochondrial Ca^2+^ uptake experiments, retinas were incubated in KRB buffer containing 100 µM KB-R7943 for 10 min. After this preincubation, baseline measurements of mito-GCaMP3 fluorescence were taken for 3 frames (30 s each) before treatment with 25 µM sildenafil for a total of 15 min of imaging.

For analysis of all GCaMP3 experiments, any cell bodies or mitochondrial clusters where the maximum fluorescence signal was completely saturated were excluded. For basal mito-GCaMP3 fluorescence analysis, mitochondrial clusters that did not respond to ionomycin treatment were not included in the analysis. Analysis was conducted blinded (masked) to sample identity. The excitation/emission wavelengths used for mito- and cyto-GCaMP3 were 488/510 nm. Timelapses were analyzed using ImageJ + Fiji software (SCR_002285) and were corrected for X–Y drift using the MultiStackReg plugin of ImageJ^59,60^. Fixed ROIs were used to quantify average fluorescence signal across the mitochondrial cluster (for mito-GCaMP3) or cell body (for cyto-GCaMP3) at every time point. Statistical analysis and figure preparation for all GCaMP experiments was performed with GraphPad v 8.4.3 (for Mac, GraphPad Software, San Diego CA, USA, www.graphpad.com).

### Mouse electrophysiology

Mice were dark adapted overnight prior to the day of experiment and were euthanized by CO_2_ incubation. Eyes were enucleated under dim red light immediately after euthanasia followed by dissection under infrared illumination. The retinas were gently detached from posterior eye cups and were stored in dark in a dish containing oxygenated Ames medium at room temperature until recording. Recordings were conducted using previously described methods^61^. The retinas were mounted photoreceptors facing up in a closed chamber and were continuously superfused with oxygenated Ames medium (Sigma) at a flow rate of 3–5 mL/min. For isolating the a-wave of ERG, 50 μM DL-AP_4_ (Tocris) and 100 μM BaCl_2_ (Sigma) were included in the Ames medium. The recording chamber was maintained at 35–36 °C and retinas were allowed to adapt to the chamber temperature for at least 15 min before experiments. Ex-vivo transretinal ERG recordings were made in scotopic conditions by presenting light flashes produced by LEDs (Thor Labs). The ERG signals were amplified using a differential amplifier (Warner Instruments), low-pass filtered at 300 Hz (Krohn Hite Corp.), digitized using digidata 1440 (Molecular Devices), and were recorded at a sampling frequency of 10 kHz using pClamp 10 software.

### Zebrafish electrophysiology

Zebrafish were approximately 7 months of age for all electrophysiology experiments. Zebrafish were briefly dark adapted (~ 30 min), before euthanasia by ice water immersion. Eyes were enucleated into Modified Salamander Ringer’s solution (110 mM NaC, 2.5 mM KCl, 1.0 mM CaCl_2_, 1.6 mM MgCl_2_, 10.0 mM HEPES, 10.0 mM Glucose) with pH adjusted to 7.8 with NaOH. The eyes were hemisected and retinas isolated from the eyecup. All procedures after the dark adaptation were performed under dim red light. To ensure ex vivo ERG signal was predominantly cone responses, dark adaption was limited to ~ 30 min to allow cone photopigment regeneration but not provide enough time for full rod photopigment regeneration, and experiments were carried out during the day (between 11 AM and 4 PM) when rod contributions to retinal responses are at their lowest due to the circadian regulation of photoreceptor biology in the zebrafish retina. Ex vivo ERG recordings were performed as described previously^61,62^. Isolated retinas were mounted photoreceptor side up onto the specimen holder and perfused with Modified Salamander Ringer’s solution, supplemented with 40 µM DL-AP4 (Tocris Bioscience) and 40 µM CNQX (Tocris Bioscience) to isolate the photoreceptor component of the ERG signal (a-wave). The rate of perfusion was ~ 5 mL/min and the experiments were conducted at room temperature (~ 23 °C). ERG signal was first amplified (100 ×) and low-pass filtered at 300 Hz by a differential amplifier (DP-311, Warner Instruments), and data was further amplified (10 ×) and acquired at 10KHz using an integrated amplifier/digitizer (IPA, Sutter Instrument, CA). A High Power LED light source (Solis-3C, Thorlabs, Newton, NJ), with filter for red light (630 nm, FWHM bandwidth 69 nm, FF01-630/69-25, Semrock, Rochester, NY) and LED driver (DC2200, Thorlabs) were used to provide the flashes of light stimuli, durations ranged from 5 to 100 ms. The SutterPatch software (SutterPatch v1.1.2, Sutter Instrument, CA) drove both stimulus generation and data acquisition via the IPA amplifier’s analogue output and input, respectively. Light stimuli were calibrated before experiments using a calibrated photodiode (FDS100-CAL, Thorlabs, Newton, NJ) and flash intensities converted to photons/µm^2^. Data analysis, including statistical analysis and figure preparation, was performed with GraphPad v 8.0.0 (for Windows, GraphPad Software, San Diego CA, USA, www.graphpad.com). Normalized responses were calculated for each retina by dividing the response amplitude data by the maximal amplitude measured at the peak/plateau of the response to the brightest flash. To quantify the gain of phototransduction activation, we fitted the Lamb–Pugh model to the initial leading edge of the dim flash response for each retina, and compared the average amplification constant (A) between WT and *mcu*^*-*/-^ siblings^63^.

## Supplementary information

Supplementary Information 1.

Supplementary Information 2.

## Data Availability

This study did not generate any new large datasets or codes.
