# Supplementary material for: Mitochondrial Calcium Uniporter (MCU) deficiency reveals an alternate path for Ca2+ uptake in photoreceptor mitochondria
Source: Sci Rep. 2020 Sep 29;10:16041. doi: 10.1038/s41598-020-72708-x (PMC7525533; doi:10.1038/s41598-020-72708-x)
Supplement: Supplementary file 1 — Supplementary Information 1. [file 41598_2020_72708_MOESM1_ESM.docx]

**SUPPLEMENTAL FIGURES**

**Supplemental Figure 1**

A. Genotyping results from four separate group crosses of *mcu*^+/-^ zebrafish. Fish were genotyped between 3-4 months of age. A chi-square test determined that the observed *mcu*^+/+^:*mcu*^+/-^:*mcu*^-/-^ ratio of 60:91:31 differed significantly from the expected 1:2:1 ratio (p=0.0098).


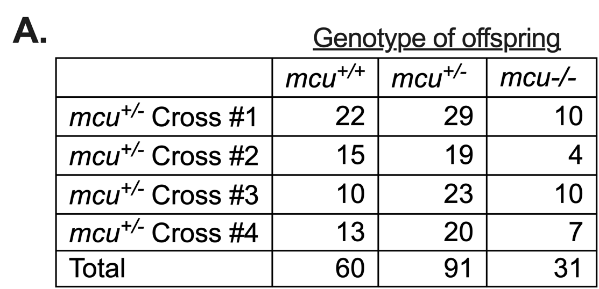


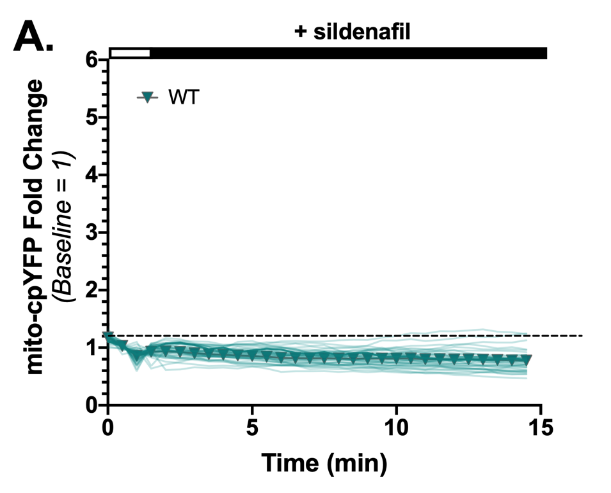


**Supplemental Figure 2**

A.  *gnat2*:mito-cpYFP (WT for Mcu expression) retina slices preincubated with 100 µM KB-R7943 (10 minutes prior to imaging and white bar) then subjected to 25 µM sildenafil (black bar). The mean response of all mitochondrial clusters is reported with the dark trace, while the semi-transparent traces show the responses of each individual mitochondrial cluster. (Mitochondrial clusters from n=48 WT mitochondrial clusters from multiple slices from 2 fish shown).


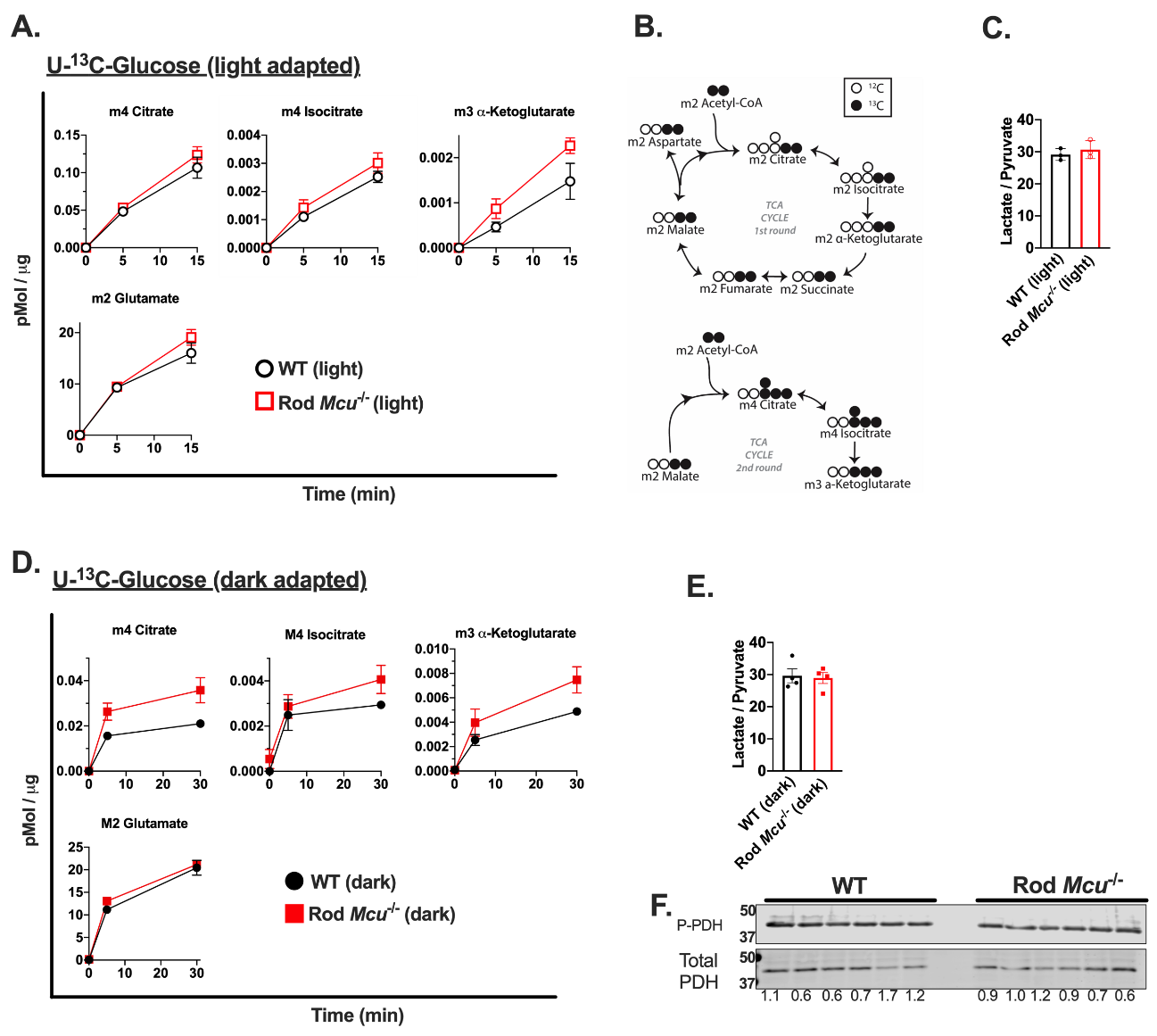


**Supplemental Figure 5**

1. Additional metabolites from the time course of light-adapted WT and Rod *Mcu*^-/-^ retinas incubated in U-^13^C-glucose for 0, 5, and 15 minutes (n=3 WT and 3 Rod *Mcu*^-/-^ retinas per time point).
2. Isotopomer diagram showing labeling pattern of TCA cycle metabolites made by retinas supplied with U-13C-glucose.
3. Lactate/Pyruvate ratio in freshly dissected light-adapted WT and Rod *Mcu^-/-^* retinas (n=3 WT and 3 Rod MCU^-/-^ retinas).
4. Additional metabolites from the time course of dark-adapted WT and Rod *Mcu*^-/-^ retinas incubated in U-^13^C-glucose for 0, 5, and 15 minutes (n=3 WT and 3 Rod *Mcu*^-/-^ retinas per time point).
5. Lactate/Pyruvate ratio in freshly dissected dark-adapted WT and Rod *Mcu^-/-^* retinas (n=3 WT and 3 Rod MCU^-/-^ retinas).
6. Western blot of WT and Rod *Mcu*^-/-^ retinas probed with PDH E1a antibody and a phospho-PDH antibody. The P-PDH/PDH ratio is 0.89 ± 0.42 -fold lower in Rod *Mcu*^-/-^ retinas (ns, mean ± standard deviation propagated to include WT error shown). Quantification of the P-PDH/PDH ratio of each sample relative to the average WT P-PDH/PDH ratio is shown below each lane (n=6 WT and 6 Rod *Mcu*^-/-^ retinas from 3 animals each).


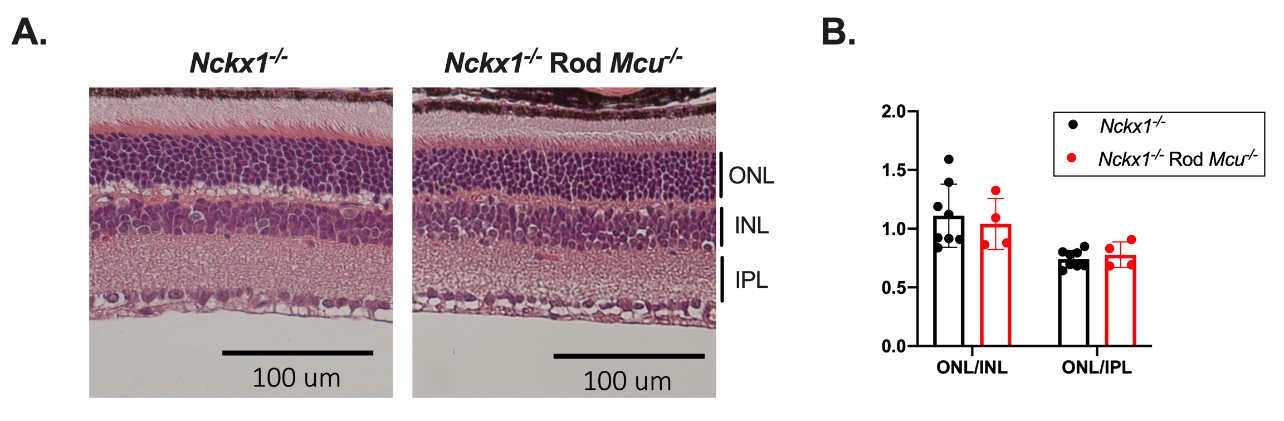


**Supplemental Figure 7**

1. Representative images of *Nckx1^-/-^* and *Nckx1^-/-^* Rod *Mcu^-/-^* hemotoxylin and eosin (H&E) stained retinal sections. Mice were between 6-8 weeks of age. Lines to the right of the *Nckx1^-/-^* Rod *Mcu^-/-^* image retinal indicate the various layers (ONL = outer nuclear layer; INL = inner nuclear layer; IPL = inner plexiform layer.
2. Quantification of H&E stained images. (n=8 eyes from four *Nckx1^-/-^* mice and 4 eyes from two *Nckx1^-/-^* Rod *Mcu^-/-^* mice).
