# Supplementary figures and images for: Mitochondrial Calcium Uniporter (MCU) deficiency reveals an alternate path for Ca2+ uptake in photoreceptor mitochondria
Source: Sci Rep. 2020 Sep 29;10:16041. doi: 10.1038/s41598-020-72708-x (PMC7525533; doi:10.1038/s41598-020-72708-x)

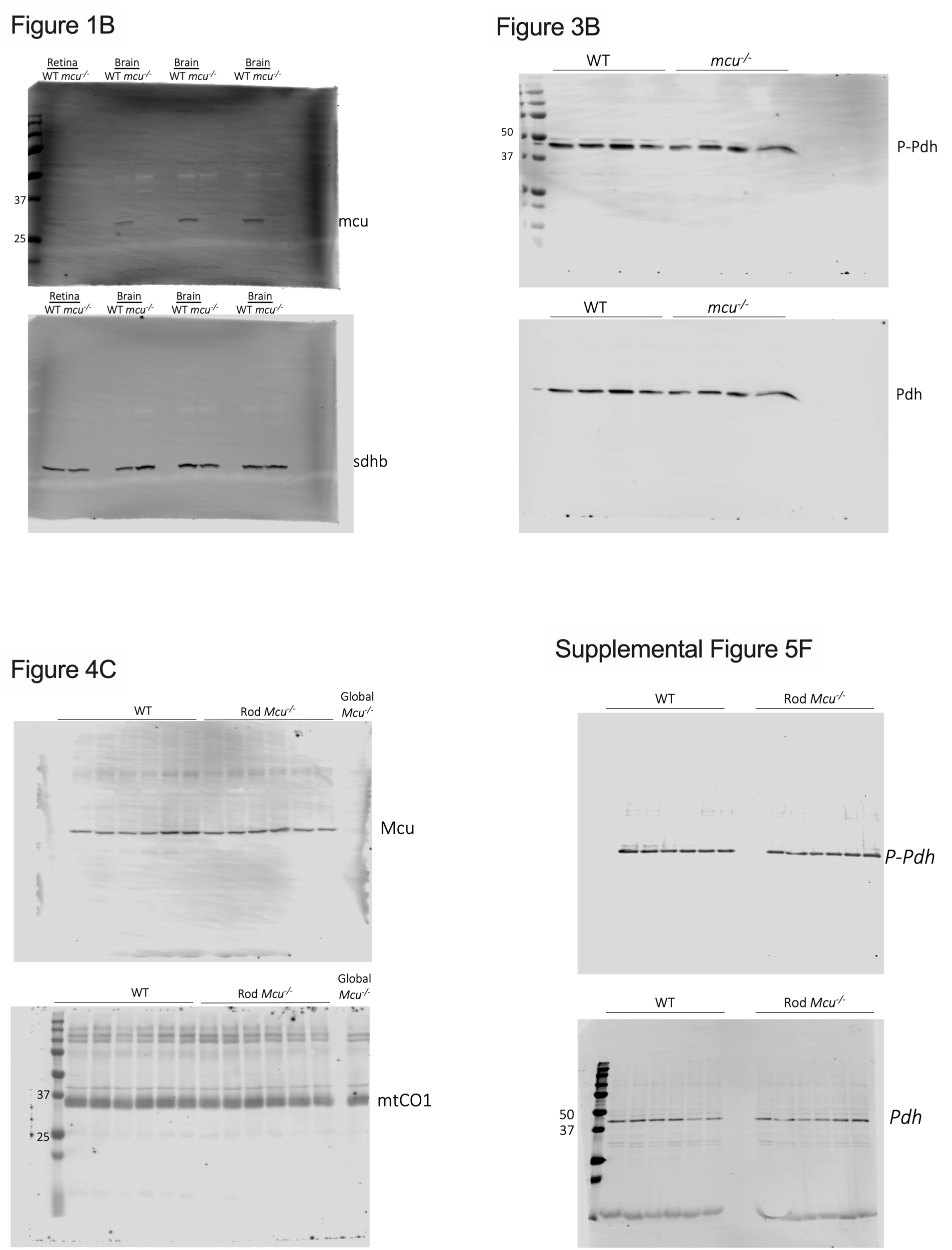

Supplement: Supplementary file 2 — Supplementary Information 2. [file 41598_2020_72708_MOESM2_ESM.tiff]
